# High efficacy of chlorfenapyr-based net Interceptor^®^ G2 against pyrethroid-resistant malaria vectors from Cameroon

**DOI:** 10.1186/s40249-023-01132-w

**Published:** 2023-08-29

**Authors:** Magellan Tchouakui, Riccado F. Thiomela, Elysee Nchoutpouen, Benjamin D. Menze, Cyrille Ndo, Dorothy Achu, Raymond N. Tabue, Flobert Njiokou, Ateba Joel, Charles S. Wondji

**Affiliations:** 1 Centre for Research in Infectious Diseases (CRID), P.O. Box 13501, Yaoundé, Cameroon; 2 Department of Animal Biology and Physiology, Faculty of Science, University of Yaoundé 1, P.O. Box 812, Yaoundé, Cameroon; 3 Department of Biological Sciences, Faculty of Medicine and Pharmaceutical Sciences, University of Douala, P.O. Box 24157, Douala, Cameroon; 4 Ministry of Public Health, National Malaria Control Programme, P.O. Box 14386, Yaounde, Cameroon; 5 Department of Vector Biology, Liverpool School of Tropical Medicine, Pembroke Place, Liverpool, L35QA UK; 6 International Institute of Tropical Agriculture (IITA), P.O. Box 2008, Yaoundé, Cameroon

**Keywords:** Malaria, *Anopheles*, Insecticide resistance, Dual active ingredient nets, Interceptor G2

## Abstract

**Background:**

The increasing reports of resistance to pyrethroid insecticides associated with reduced efficacy of pyrethroid-only interventions highlight the urgency of introducing new non-pyrethroid-only control tools. Here, we investigated the performance of piperonyl-butoxide (PBO)-pyrethroid [Permanet 3.0 (P3.0)] and dual active ingredients (AI) nets [Interceptor G2 (IG2): containing pyrethroids and chlorfenapyr and Royal Guard (RG): containing pyrethroids and pyriproxyfen] compared to pyrethroid-only net Royal Sentry (RS) against pyrethroid-resistant malaria vectors in Cameroon.

**Methods:**

The efficacy of these tools was firstly evaluated on *Anopheles gambiae* s.l. and *Anopheles funestus* s.l. from Gounougou, Mibellon, Mangoum, Nkolondom, and Elende using cone/tunnel assays. In addition, experimental hut trials (EHT) were performed to evaluate the performance of unwashed and 20 times washed nets in semi-field conditions. Furthermore, pyrethroid-resistant markers were genotyped in dead vs alive, blood-fed vs unfed mosquitoes after exposure to the nets to evaluate the impact of these markers on net performance. The XLSTAT software was used to calculate the various entomological outcomes and the Chi-square test was used to compare the efficacy of various nets. The odds ratio and Fisher exact test were then used to establish the statistical significance of any association between insecticide resistance markers and bed net efficacy*.*

**Results:**

Interceptor G2 was the most effective net against wild pyrethroid-resistant *An. funestus* followed by Permanet 3.0. In EHT, this net induced up to 87.8% mortality [95% confidence interval (*CI*): 83.5–92.1%) and 55.6% (95% *CI*: 48.5–62.7%) after 20 washes whilst unwashed pyrethroid-only net (Royal Sentry) killed just 18.2% (95% *CI*: 13.4–22.9%) of host-seeking *An. funestus*. The unwashed Permanet 3.0 killed up to 53.8% (95% *CI*: 44.3–63.4%) of field-resistant mosquitoes and 47.2% (95% *CI*: 37.7–56.7%) when washed 20 times, and the Royal Guard 13.2% (95% *CI*: 9.0–17.3%) for unwashed net and 8.5% (95% *CI*: 5.7–11.4%) for the 20 washed net. Interceptor G2, Permanet 3.0, and Royal Guard provided better personal protection (blood-feeding inhibition 66.2%, 77.8%, and 92.8%, respectively) compared to pyrethroid-only net Royal Sentry (8.4%). Interestingly, a negative association was found between *kdrw* and the chlorfenapyr-based net Interceptor G2 (*χ*^2^ = 138; *P* < 0.0001) with homozygote-resistant mosquitoes predominantly found in the dead ones.

**Conclusions:**

The high mortality recorded with Interceptor G2 against pyrethroid-resistant malaria vectors in this study provides first semi-field evidence of high efficacy against these major malaria vectors in Cameroon encouraging the implementation of this novel net for malaria control in the country. However, the performance of this net should be established in other locations and on other major malaria vectors before implementation at a large scale.

**Graphical Abstract:**

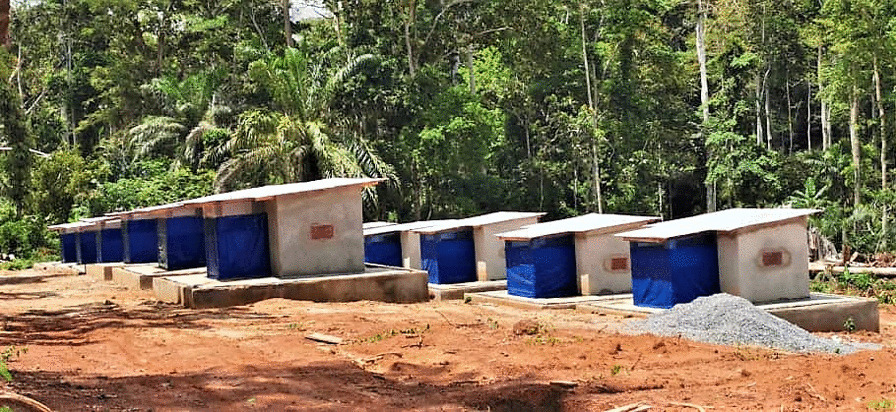

**Supplementary Information:**

The online version contains supplementary material available at 10.1186/s40249-023-01132-w.

## Background

The burden of malaria disease in Africa is still alarming despite the significant progress achieved in the past two decades [1]. The major driver of the reduction of malaria incidence has been attributed to the large-scale implementation of long-lasting insecticide-treated nets (LLIN) and indoor residual spraying (IRS) as reported by several studies conducted across the continent [2–4]. Bednets accounted for more than 68% of the reduction in malaria incidence. Pyrethroids remain the insecticides of choice for impregnating the nets due to their low cost, fast-acting effect, longer residual activity, and safety [5–7]. Unfortunately, the escalation of pyrethroid resistance in major malaria vectors is jeopardizing the success of control programs relying on this insecticide class [8, 9]. Resistance to pyrethroids has now been reported in all major malaria vectors in 27 countries across sub-Saharan Africa including Cameroon [10, 11].

Target-site (knockdown resistance; L1014F/S-kdr) and metabolic resistance are the main mechanisms driving this resistance in *Anopheles gambiae* s.l [12]. whereas metabolic resistance driven mainly by cytochrome P450s is the main driver of pyrethroid resistance in *Anopheles funestus* with no evidence of knock-down resistance (*kdr)* [13–15]. The widespread pyrethroid resistance with evidence of reduced efficacy on pyrethroid-only nets [4, 13, 16, 17] has led to the design of new generation nets to combat this threat. The first wave of these new nets included insecticide-treated nets (ITNs) incorporating piperonyl butoxide (PBO), a synergist that inhibits cytochrome P450s. Novel insecticides introduced by manufacturers and recommended by World Health Organization (WHO) for ITNs include chlorfenapyr, a repurposed pyrrole from agriculture used in new generation nets such as Interceptor G2 (IG2), and pyriproxyfen, a growth regulator used in the Royal Guard (RG) net [18]. Testing the efficacy of these new tools on the field population of malaria vectors and assessing the impact of known resistance markers such as *kdr* on their performance is highly needed before implementation at a large scale.

Several experimental hut trials (EHTs) have previously shown the efficacy of these new-generation nets [19–22]. Moreover, recent randomized control trials (RCTs) in Tanzania have shown the greater efficacy of Interceptor G2 over PBO [23] and now in Benin [24]. However, because resistance varies in intensity and mechanisms according to vector species and geographical location, the National Malaria Control Program (NMCP) needs evidence to make the appropriate choice of nets most suitable in their context. That was the case for Cameroon NMCP which by 2019 was engaged in planning the next phase (2022–2023) of mass distribution of LLINs nationwide. Having solely distributed pyrethroid-only nets in previous campaigns, the NMCP needed evidence of which nets would be most suitable for Cameroon. Some bioefficacy testing and EHTs performed had revealed a drastic loss of efficacy of conventional nets across the country although PBO nets induced significantly higher mortality against pyrethroid-resistant *An. funestus* than pyrethroid-only nets [25]. However, it was also shown that the efficacy of the PBO-based net (Olyset plus) was impacted by glutathione-S-transferase (GST)-mediated metabolic resistance which is not affected by the synergistic action of PBO [25]. Such loss of efficacy of pyrethroid-only nets underscores the urgent need for alternative tools or new insecticide formulations to complement existing ones and preserve LLINs effectiveness. Due to a lack of data to guide the NMCP in Cameroon in its choice of LLINs, a Technical Vector Control Advisory Group (TVCAG), established in Cameroon in 2018 through the Partnership for Increasing the Impact of Vector Control (PIIVEC) program, called for a study to establish the situation in Cameroon in a collaboration between NMCP and the Centre for Research in Infectious Diseases (CRID). This study sought to assess the efficacy of Interceptor^®^ G2, a net with a mixture of chlorfenapyr and alpha-cypermethrin, in an area where pyrethroid nets have limited efficacy on malaria vectors.

## Methods

### Study sites

The mosquitoes used in this study include *An. funestus* from Mibellon (Adamaoua) and Elende (Centre), *An. gambiae* s.l*.* from Mangoum (West) and Nkolondom, (Centre), and *An. coluzzi* from Gounougou (North) collected between May and October 2021. The choice of these sites was based on the stratification of NMCP for the 2022–2023 nationwide campaign of bednet distribution.

**Mibellon** is a rural village located in Central-northern Cameroon (Adamaoua region). The climate is Sudano-Guinean characterized by an eight-month rainy season from March to October and a dry season of four months extending from November to February [21]. The village is located close to permanent water bodies, including a lake and swamps, which provide suitable breeding sites for mosquito development. *An. gambia*e s.l. and *An. funestus* s.l. are the two main malaria vectors found in the village, with *An. funestus* the most abundant throughout the year with a greater role in malaria transmission (792 infective bites/person per year) [26]. *An. funestus* populations from Mibellon display a high resistance intensity to all the pyrethroids which has led to a loss of efficacy of pyrethroid-based LLINs [25].

**Elende** (3°41′57.27″N, 11°33′28.46″E) is a rural village situated in central Cameroon close to Yaoundé. This village is characterized by a classical Guinean equatorial climate with four distinct seasons: a short rainy season from mid-March to the end of June; a short dry season from late June to mid-August; a long rainy season that runs from mid-August to mid-November and a long dry season which runs from mid-November to mid-March. This locality is highly endemic to malaria (350 infective bites/per person per year) [26] with *An. funestus* as the predominant vector species.

**Mangoum** (5°29′09.2″N, 10°35′20.8″E) is located in the western part of the country and characterised by extensive agricultural practices and four seasons: two rainy seasons (March–June and September–November) and two dry seasons (December–February and July–August). *An. gambiae* s.s. is predominant in Mangoum with 4.1% *Plasmodium* infection rate*.* This species is resistant to the four main classes of public health insecticides, with mortality rates < 50% at 10 × the pyrethroid discriminating doses [27].

**Nkolondom** (3°57′18″N, 11°29′36″E) is situated in the Centre region and the climate is characterized by two wet and two dry seasons. The rains mostly fall from March to November, with two peaks in May (219.7 mm) and October (296.1 mm) and a relative breakdown between July and August (short dry season) when precipitation does not exceed 120 mm per month. The main dry season lasts for 3 months, from December to February; the rainfall at that time is less than 50 mm per month. *An. gambiae* s.s. is predominant in in this location as in Mangoum and is resistant to the four main classes of public health insecticides [28]

**Gounougou** (9.07°N, 13.55°E) is a rice cultivation area located in the dry savannah zone of the North. The area has a rainy season of about 6 months from May to October. The predominant vector in this area is *An. coluzzii* followed by *An. funestus,* but also *An. arabiensis* and other local vectors such as *An. pharoensis*. *An. coluzzii* from the location are highly resistant to permethrin, deltamethrin and DDT with loss of efficacy of bednet efficacy [29].

### Mosquito’s collection for cone/tunnel

Two different methods were used for mosquito collection. The dipping method was used to collect immature stages of *An. gambiae* s.l. in the breeding sites at Mangoum, Nkolondom, Elende, and Gounougou. The collected immature stages were reared at the CRID insectary and the emerging adults (2–5 days old) were used for the testing. *An. funestus* mosquitoes were sampled at the adult stage (indoor resting female) at Mibellon and Elende using electric aspirators. They were forced to lay eggs and larvae were reared till the adult stage (F_1_). Two-five days old female mosquitoes (F_1_) from the collected adults (F_0_) were used for cone/tunnel assays.

### Net treatments for cone/tunnel tests and experimental hut trials

The nets below (Table 1) sourced directly from manufacturer were used for the evaluation.

**Table 1:** List of net brands used in Cone/tunnel tests and experimental hut trials

Net brands	Composition	Material	Experiment
Control	Untreated	Polyester	Cone test and EHT
Royal Sentry	Alpha-cypermethrin (261 mg/m^2^)	Polyethylene	Cone test and EHT
Royal Guard	Alpha-cypermethrin (208 mg/m^2^) + Pyriproxyfen (208 mg/m^2^)	Polyethylene	Cone test and EHT
Interceptor	Alpha-cypermethrin (200 mg/m^2^)	Polyester	Cone test and EHT
Interceptor G2	Alpha-cypermethrin (100 mg/m^2^) + Chlorfenapyr (200 mg/m^2^)	Polyester	Tunnel test and EHT
Olyset	Permethrin (1000 mg/m^2^)	Polyethylene	Cone test
Olyset plus	Permethrin + 1% PBO (1000 mg/m^2^)	Polyethylene	Cone test
Permanet 2.0	Deltamethrin (55 mg/m^2^)	Polyester	Cone test
Duranet	Alpha-cypermethrin (261 mg/m^2^)	Polyethylene	Cone test
PermaNet 3.0	Deltamethrin (84 mg/ m^2^ on the sides and 120 mg/m^2^ + 800 mg/m^2^ PBO on the top)	Polyester	Cone test and EHT

*PBO* Piperonyl butoxide; *EHT* experimental hut trial

### Cone and tunnel assay procedure

Cone assays were performed for the unwashed standard net and new ITNs except the interceptor G2 for which tunnel test has been performed. Cone tests were carried out using five pieces of the net (30 cm × 30 cm). Two replicates of five susceptible or field-resistant mosquitoes were exposed for 3 min. After exposure, the mosquitoes were removed from the cones using a mouth aspirator and then transferred into paper cups, and fed with 10% sugar solution. Numbers of mosquitoes knocked down were recorded after 60 min. A negative control (untreated net) was included in each of the LLIN cone test. Post-exposure mortality was recorded after 24 h of observation under standard insectary conditions (temperature 25 °C ± 2 °C and 80% ± 10% relative humidity).

Tunnel tests were performed for interceptor G2 as this net contains a slow-acting insecticide (chlorfenapyr). A total of 100 sugar-starved (for 1 h) mosquitoes aged 5–8 days were released in the long section of the glass tunnel at 06:00 pm. A guinea pig was used as bait and positioned on the other side of the net so that mosquitoes must pass through the holed net to access the bait and feed. The following morning, between 06:00 and 09:00 am, mosquitoes were removed (separately from each section of the tunnel) using a mouth aspirator, counted, and scored as alive or dead, blood-fed or unfed after which they were held for 72 h with access to 10% sugar solution at 27 °C ± 2 °C and 80% ± 10% relative humidity. The main outcome measures were 12 h mortality measured in the morning after the experiment, 72 h post-exposure mortality, and blood feeding inhibition [30].

### Impact of pyrethroid-resistance markers on bed nets efficacy after cone/tunnel assays

As the *kdr* is fixed in all the *An. gambiae* s.l. populations tested, we crossed the highly resistant pyrethroid-resistant strain from Nkolondom with the susceptible lab strain KISUMU to have the hybrid strain NKOL/KIS with the segregated genotype for comparisons. More specifically, larvae/pupae collected at Nkolondom were reared till the pupal stage which was isolated individually in 15 ml falcon tubes and after emergence, all males were mixed in cages containing the female KISUMU. After crosses between females KISUMU and males from Nkolondom, the progeny obtained were left in cages for intercrosses for four generations and the hybrid of the 4th generation (F_4_) was used for cone and tunnel assays. After the tests, the alive and dead mosquitoes were genotyped for the L1014F-kdr mutation.

### Experimental hut trials

Experimental hut trials were conducted in Elende (3°41′57.27″N, 11°33′28.46″E) to evaluate the performance of PBO-pyrethroid and dual AI nets compared to pyrethroid-only net on the local free-flying malaria vectors in semi-field condition.

### Design of the experimental huts

Nine huts were constructed from concrete bricks, with a corrugated aluminum roof, and a concrete base surrounded by a water-filled channel to prevent the entry of ants according to the WHO guidelines [30]. These huts were designed in the West Africa region style with mosquito access occurring via four window slits constructed from pieces of metal, fixed at an angle to create a funnel with a 1-cm wide gap. Mosquitoes fly upward to enter through the gap and then downwards to exit into the hut; this precludes or greatly limits their exit through the aperture enabling most of the entering mosquitoes to be accounted for. A single veranda trap made of polyethylene sheeting and screening mesh measuring 2-m long, 1.5-m wide, and 1.5-m high, projects from the back wall of each hut. The movement of mosquitoes between the hut and veranda is unimpeded during the night.

### Washing procedure of nets

The washing solution (2 g soap/liter) was prepared by adding 20 g Marseille soap to 10 L of dechlorinated water contained in the aluminum washing tank. The net was washed daily for 10 min. Once immersed in the washing solution, it underwent two manual rotation periods of 20 rpm for 3 min interspersed with 4 min of holding. The net was rinsed following the same steps as the washing using dechlorinated water.

### Sleepers and mosquito collection

Adult volunteers slept under the nets and mosquitoes were collected the following day. The volunteers were recruited among the inhabitants of Elende after reading and signing the informed consent. The treatments and sleepers were randomly allocated to 9 experimental huts and rotated for 36 nights according to the Latin Square design (sleepers are rotated daily, and treatments are rotated weekly) to adjust for any difference in the hut and individual attractiveness. Volunteers started collecting mosquitoes in the nets and the different compartments of the huts in the morning from 5:30 am. Mosquitoes were brought to the local laboratory, for species identification, mortality recording, and determination of blood-feeding status. Live mosquitoes were put in small cups netted with plastic and provided 10% sugar solution for 24 h and 72 h (Interceptor G2) to assess delayed mortality. Mosquitoes were collected over six weeks between December 2021 and January 2022. The following outcomes were measured: (i) deterrence (reduction in hut entry relative to the control huts fitted with untreated nets); (ii) induced exophily (the proportion of mosquitoes found in the veranda relative to the total collected mosquitoes); (iii) blood-feeding inhibition (the reduction in blood-feeding of mosquitoes relative to the control huts); (iv) immediate and delayed mortality (the proportion of dead mosquitoes when collected and after 24/72 h); (v) personal protection: the reduction in mosquito biting by treated nets relative to untreated nets.

### Association between pyrethroid resistance markers and bed nets efficacy after EHT

To establish the impact of insecticide resistance to pyrethroids on the effectiveness of PBO/dual AI nets, *An. funestus* mosquitoes of each treatment including the dead, alive, blood-fed, and unfed mosquitoes from EHT were genotyped for L119F-GSTe2 marker previously shown to confer pyrethroid resistance in *An. funestus* [31].

### Data analysis

The XLSTAT software (Addinsoft, Berkeley, CA, USA) was used to calculate the various entomological outcomes described above and the chi-square test incorporated in this software helped to compare these outcomes between different treatments relative to the control as done previously [32, 33]. The number of mosquitoes collected in the huts with different treatments was analysed by negative binomial regression and the effect of each treatment on the main entomological outcomes (exophily, blood feeding, and mortality) was assessed using binomial generalized linear mixed models (GLMMs) with a logit link function, fitted using the ‘lme4’ package for R 3.6 as shown [25]. A separate model was fitted for each outcome and each model included random effects to account for the following sources: the nine huts used; the nine sleepers and the six weeks of the trial. The efficacy of standard and PBO/dual AI nets was evaluated by comparing the mean mortality rate obtained between different categories of nets with the Chi-square test. Odds ratio and Fisher exact test were then used to establish the statistical significance of any association between *kdr* and the ability of mosquitoes to survive exposure to various bednets as done previously for other markers [13, 16, 17, 34].

## Results

### Bio-efficacy of insecticides treated net against field-resistant mosquitoes using cone and tunnel test

**Fig. 1:**
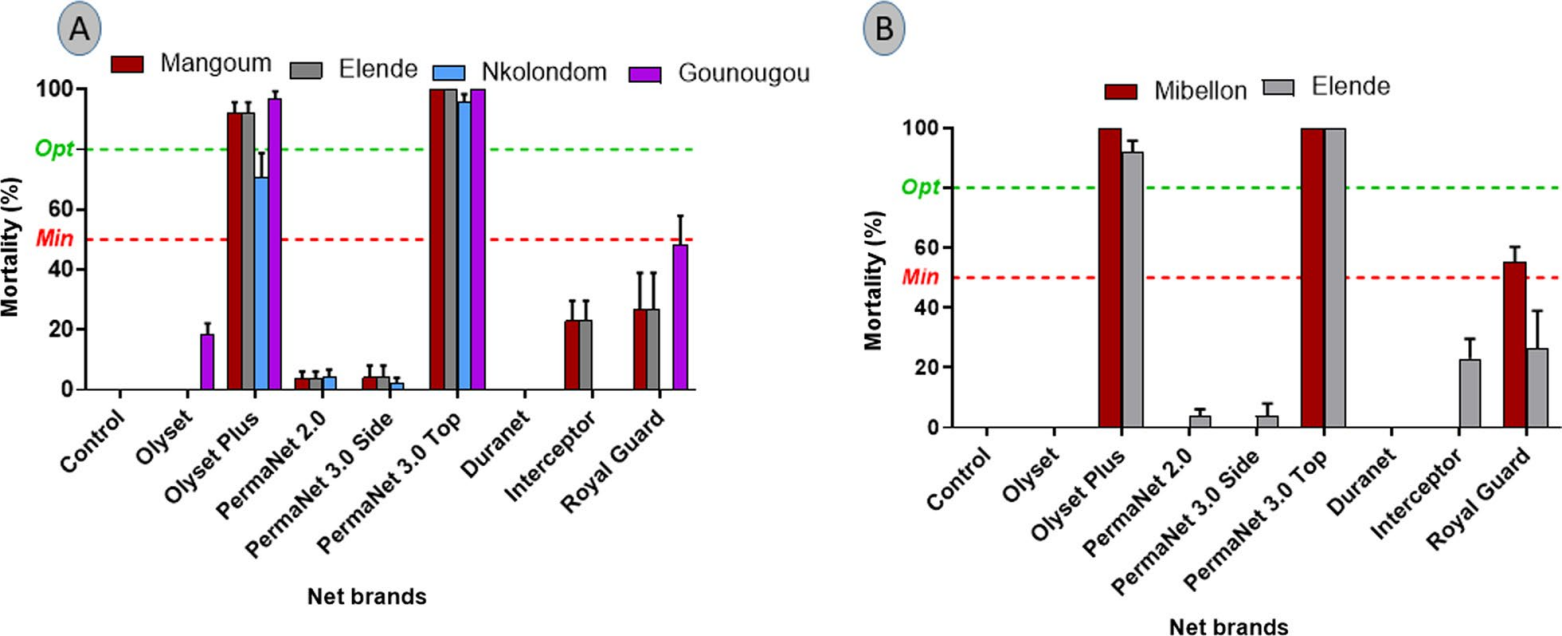
Performance of PBO-based and new generation nets compared to pyrethroid-only nets against *Anopheles gambiae* s.l. and *Anopheles funestus* s.l. in cone test. Mortality rate 24 h after 3-min cone test for various nets against wild *Anopheles gambiae* s.l. (**A**), and wild *Anopheles funestus* s.l. (**B**); Red dot line represents minimal efficacy (50% mortality) and the green dot represents optimal efficacy (80% mortality) of the nets. Error bars represent the standard error on the mean (SEM)

High mortality (> 90%) was noticed with the KISUMU strain for all the nets in the cone test confirming their good quality (Additional file 1: Fig. S1). However, a low efficacy of standard nets (Olyset and PermaNet 2.0, interceptor, and Duranet) was observed against all the *An. gambiae* s.l. (Fig. 1A) and *An. funestus* s.l. (Fig. 1B) field populations with a mortality rate of less than 20%. PBO-based nets (OlysetPlus, and PermaNet 3.0) induced the highest mortality in all the populations compared to other nets (mortality rate ≥ 80% except in Nkolondom where a reduced efficacy to olyset plus was observed) (Fig. 1A). For dual active ingredient (AI) nets, very low mortality was recorded against Royal Guard against all resistant populations (Fig. 1A, B).

**Fig. 2:**
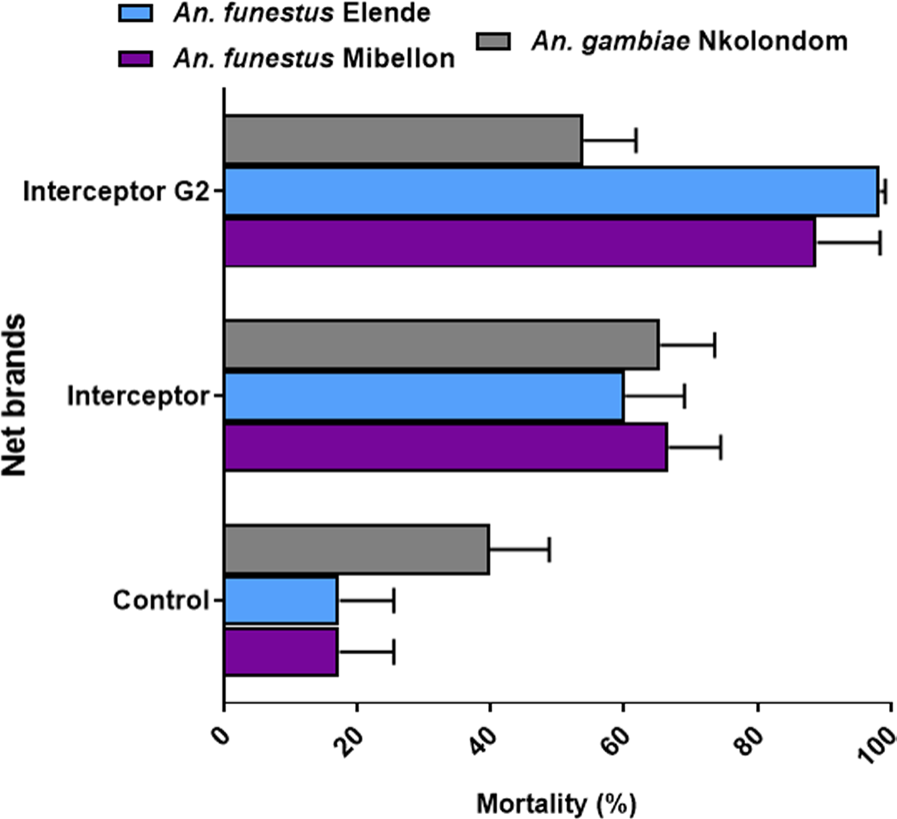
Performance of the dual AI net interceptor G2 compared to pyrethroid-only net interceptor against *Anopheles gambiae* s.l. and *Anopheles funestus* s.l. in tunnel test. Mortality rate 72 h after tunnel assay with 3 replicates of 100 mosquitoes (*n* = 300) for various nets against. Error bars represent the confidence interval on the percentage mortality

The chlorfenapyr-based net Interceptor G2 induced very high mortality after the tunnel test on *An. funestus* from Elende (mortality rate = 98.4%) and Mibellon (mortality rate = 88.9%) but reduced efficacy on *An. gambiae* s.l. from Nkolondom (mortality rate = 53.9%) (Fig. 2).

### Influence of the L1014F-*kdrw* mutation on the efficacy of LLINs tested using samples from cone/tunnel assays

**Fig. 3:**
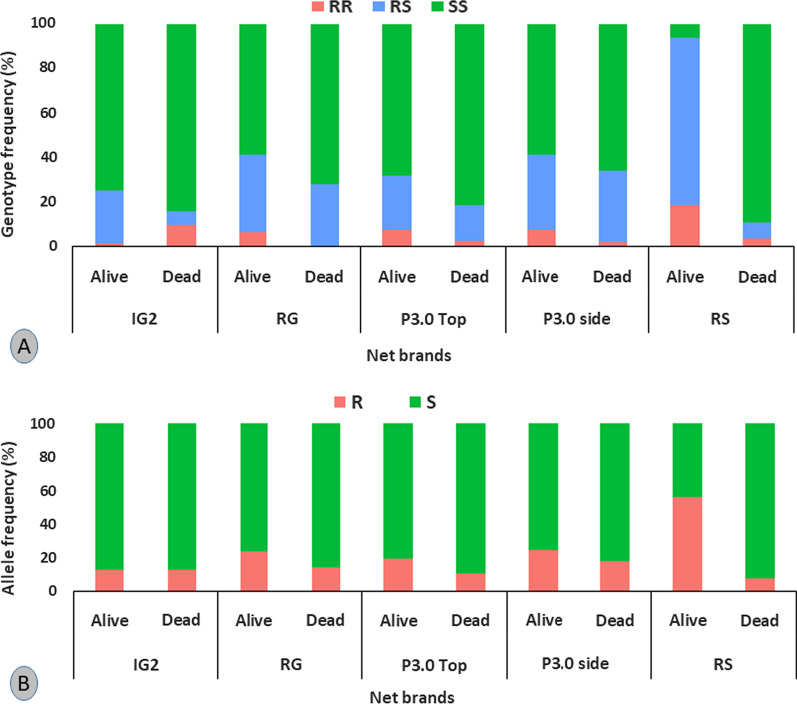
Influence of the L1014F-*kdrw* resistant marker on the efficacy of bed nets after cone assays with hybrid *Anopheles gambiae* s.l. Genotype (**A**) and allele (**B**) distribution of L1014F mutation between alive and dead *An. gambiae* s.l. after exposure to Interceptor G2, Royal Guard, Permanet 3.0, and the standard net Royal Sentry. IG2: interceptor G2, RG: Royal Guard, P3.0: Permanet 3.0, RS: Royal Sentry. For genotype, RR: homozygote resistant, RS: heterozygote, SS: homozygote susceptible. In **B**, R: resistant allele, S: susceptible allele

The L1014F-*kdrw* mutation marker was genotyped on NKOL/KIS strain from cone/tunnel test to assess the influence of this marker on the efficacy of various nets. Homozygous resistant mosquitoes (RR) and heterozygous (RS) were significantly more able to survive exposure to the pyrethroid-only net Royal Sentry (*χ*^2^ = 138; *P* < 0.0001) compared to homozygote susceptibility (SS) (Fig. 3A, B). Similarly, no association was found between *kdr* and the ability of mosquitoes to survive exposure to Permanet 3.0 side (*P* = 0.2) and top (*P* = 0.06). However, individuals with RR genotype were mainly found among the alive mosquitoes whereas the SS were mainly found among the dead for this PBO-based net. For the novel net Royal Guard, RR mosquitoes were significantly found among the alive (*P* = 0.006) and SS more predominant among the dead showing that this new generation net could rapidly loss efficacy in areas where resistance is mainly driven by *kdr*. Interestingly, a negative association was found between *kdr* and the chlorfenapyr-based net Interceptor G2 (*χ*^2^ = 138;* P* < 0.0001) with RR individuals most found among the dead mosquitoes while the SS are predominantly among the alive mosquitoes. Odd-ratio (*OR*) analysis revealed that RS has a greater chance to survive Interceptor G2 exposure compared to RR (*OR* = 40; 95% *CI:* 4.2–376; *P* < 0.0001) and SS (*OR* = 4.5; 95% *CI*: 1.7–11.5; *P* < 0.0001). Also, SS had a better chance to survive than RR (*OR* = 8.9; 95% *CI*: 1.1–71.1; *P* = 0.01) showing that mosquitoes with the *kdr*-resistant allele are more susceptible to this net.

### Experimental hut results

**Fig. 4:**
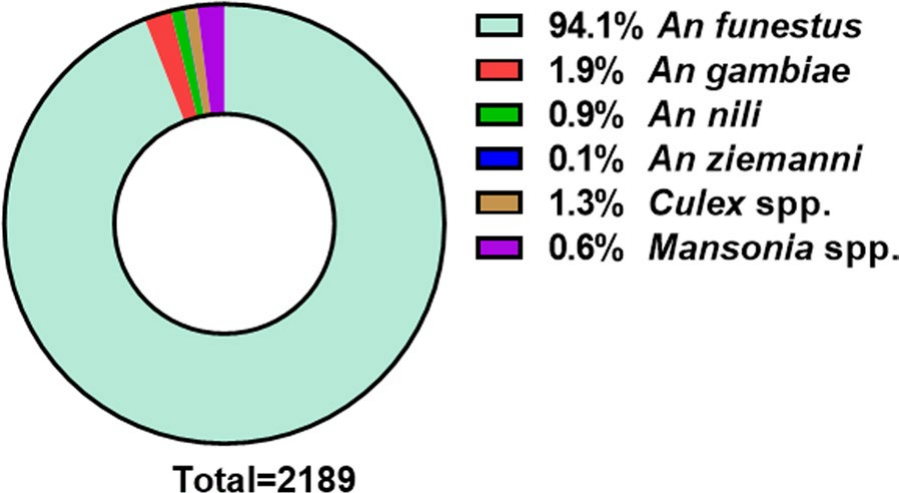
Composition of mosquitoes collected during the experimental hut trials in Elende from December 2021 to January 2022

A total of 2189 mosquitoes were collected from the 9 experimental huts during the study period and *An. funestus* s.l. was predominant at 94.1% (males = 108, and females = 1952). Among other *Anopheles* species collected, we had 21 (0.9%) *An. nili*, 43 (1.9%) *An. gambiae* s.l. and 1 *An. ziemanni*. The culicinae collected included 43 *Mansonia* spp. (0.6%) and 21 (1.3%) *Culex* spp (Fig. 4).

### Deterrence effect of the net tested

Analysis of the deterrence effect of these nets on *An. funestus* s.l., no significant difference was observed in the mean number of mosquitoes collected in unwashed treated huts compared to untreated nets suggesting that the deterrence effect is not impacted by the washing except for royal guard where 20-washed net had more mosquitoes than unwashed (*n* = 253 for unwashed and 367 for 20 washes) (Additional file 2: Table S1). Chlorfenapyr-based net interceptor G2 unwashed had 221 mosquitoes whereas 187 mosquitoes were collected with nets 20 times washed. The deterrence effect of Permanet 3.0 was not impacted by the washing (*n* = 104 for unwashed and 106 for 20 washes) same for the standard net Royal Sentry (*n* = 253 for unwashed and 248 for 20 washes). Overall, Permanet 3.0 huts had a lower entry rate compared to other nets probably linked to its high repellency effect.

### Induced exophily

**Fig. 5:**
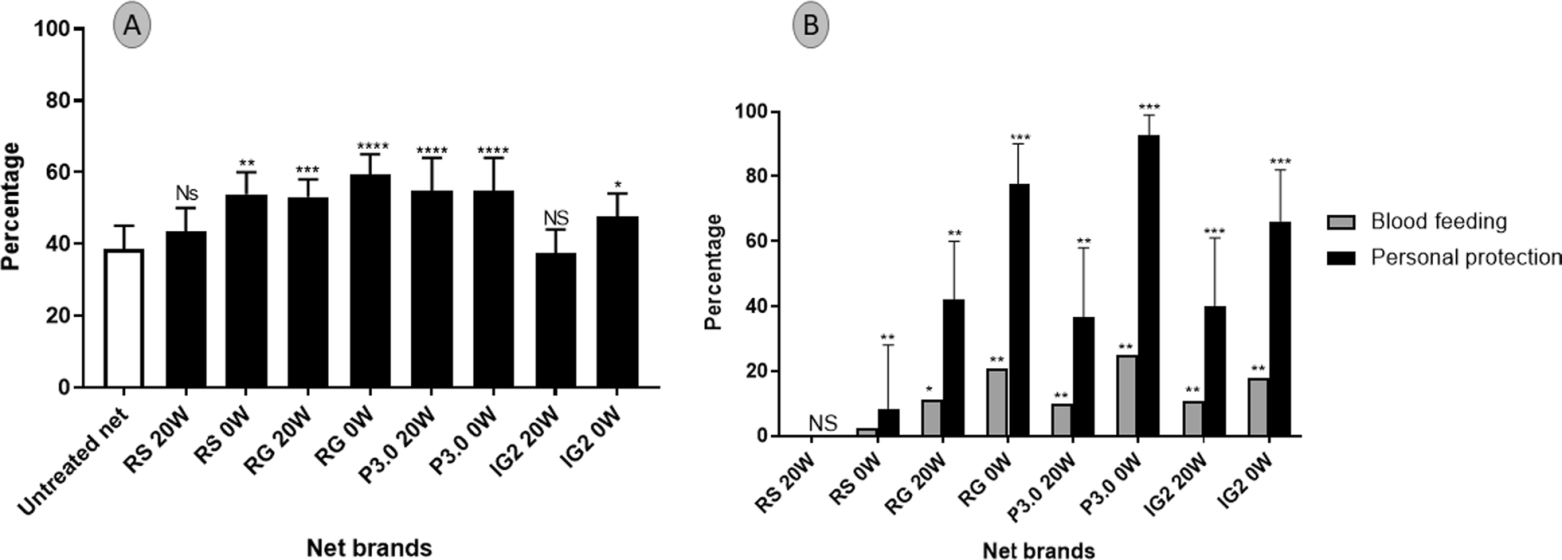
Exophilic rate (**A**) and blood-feeding inhibition with personal protection rate (**B**) during the experimental huts trials in Elende. Error bars represent the 95% confidence interval and * represents the level of significance for each LLIN relative to the control net and personal protection rate (PPR). IG2: interceptor G2, RG: Royal guard, P3.0: Permanet 3.0, RS: Royal Sentry, 0w: unwashed nets, 20w: 20 times-washed nets

Compared to untreated nets, all the treated unwashed nets had higher exophilic rates: Interceptor G2 (*χ*^*2*^ = 3.6; *P* = 0.05), Royal Guard (RG) (*χ*^*2*^ = 19.9; *P* < 0.0001), Permanet 3.0 (P3.0) (*χ*^*2*^ = 7.5; *P* = 0.006), and Royal Sentry (RS) (*χ*^*2*^ = 17.11; *P* < 0.0001) (Fig. 5A). The exophilic rate remained significantly high after 20 washes for the dual AI net Royal Guard (*χ*^*2*^ = 11.5; *P* = 0.0007), and the PBO net Permanet 3.0 (*χ*^*2*^ = 7.5; *P* = 0.006) compared to the untreated net but did not differ for the dual AI. Interceptor G2 (*χ*^*2*^ = 0.05; *P* = 0.8) and the standard net Royal Sentry (*χ*^*2*^ = 1.2; *P* = 0.3). The Interceptor G2 induced the lower exophilic rate probably due to the low quantity of alphacypermethrin in this net and the non-repellency property of chlorfenapyr. Overall, the exophily was not influenced by the hut and sleepers as well as the week except for week 5 and week 6 where the exophily was higher (*P* < 0.05) (Additional file 3: Table S2a).

### Blood-feeding inhibition

Blood feeding inhibition of *An. funestus* s.l. mosquitoes relative to control (untreated net) was higher for the dual net Royal Guard (*χ*^2^ = 19.3; *P* < 0.0001) and Interceptor G2 (*χ*^2^ = 20.8; *P* < 0.0001), and the PBO net Permanet 3.0 (*χ*^2^ = 28.7; *P* < 0.0001) resulting in a better personal protection rate compared to other nets (Fig. 3B). The pyrethroid-only net Royal Sentry produced similar blood-feeding inhibition compared to the control (*χ*^2^ = 1.1; *P* = 0.3). The blood feeding inhibition was reduced for all the nets after 20 washes but not significantly compared to unwashed (Fig. 4B). Contrary to the exophily, the blood-feeding varied significantly according to the weeks (Additional file 3: Table S2b).

### Mortality rate

**Fig. 6:**
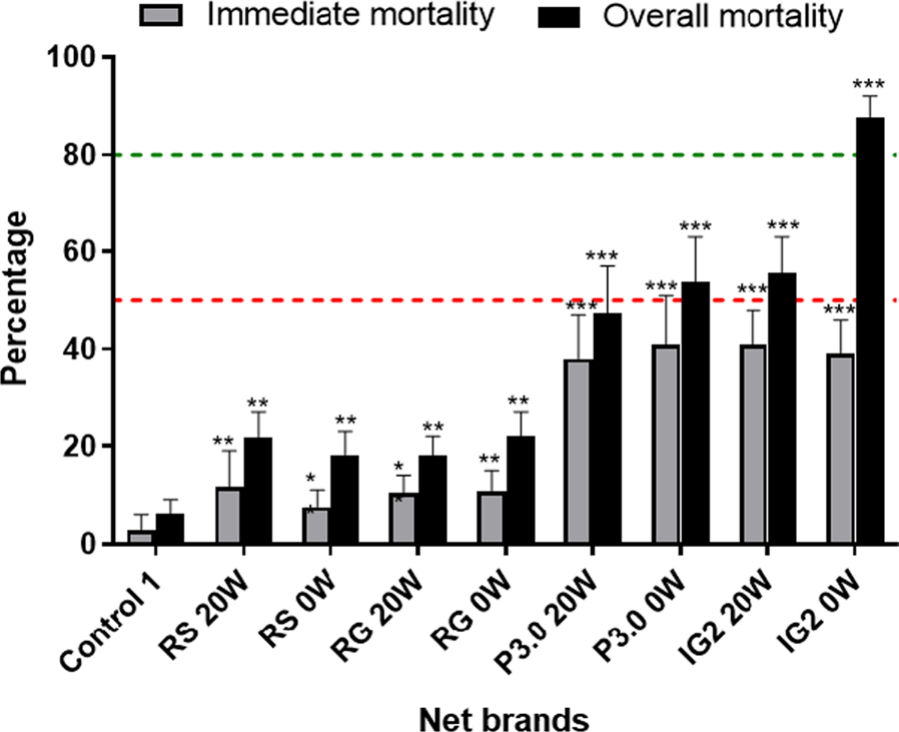
Percentage mortality of *Anopheles funestus* s.l. corrected for control mortality 24 h and 72 h after exposure during the experimental hut trials in Elende. The red dot line represents minimal efficacy and the green dot represents the optimal efficacy of the nets. *represents the level of significance (*P < 0.05 and **P < 0.01 and ***P < 0.001). 0w: unwashed nets, 20w: 20 times-washed nets

Interceptor^®^ G2 unwashed and washed 20 times induced a higher mortality rate on *An. funestus* s.l. from Elende (87.8% for 0 wash and 57.7% for 20 washes) compared to all the other nets (Fig. 6). The PBO net Permanet 3.0 was the second most efficient net compared to others with a mortality rate of 58.8% and 47.2% for unwashed and washed 20 times respectively. The mortality rate of the pyrethroid-only net Royal Sentry and the dual net Royal Guard was not different compared to untreated net indicating a loss of efficacy of these nets in killing this local *An. funestus* s.l. population. Mortality was not found to be affected by sleepers, huts as well as the week of the study (Additional file 3: Table S2c).

### Assessment of the association between the L119F*-GSTe2* and the efficacy of dual AI and PBO-based nets after experimental hut trial

Genotyping of the *L119F-GSTe2* marker allowed us to assess the impact of GST-based metabolic resistance on the efficacy of dual AI nets (Interceptor G2 and Royal Guard), PBO-based net (P3.0) compared to pyrethroid-only net Royal Sentry.

### Influence of L119F*-GSTe2 on* mosquito mortality

**Fig. 7:**
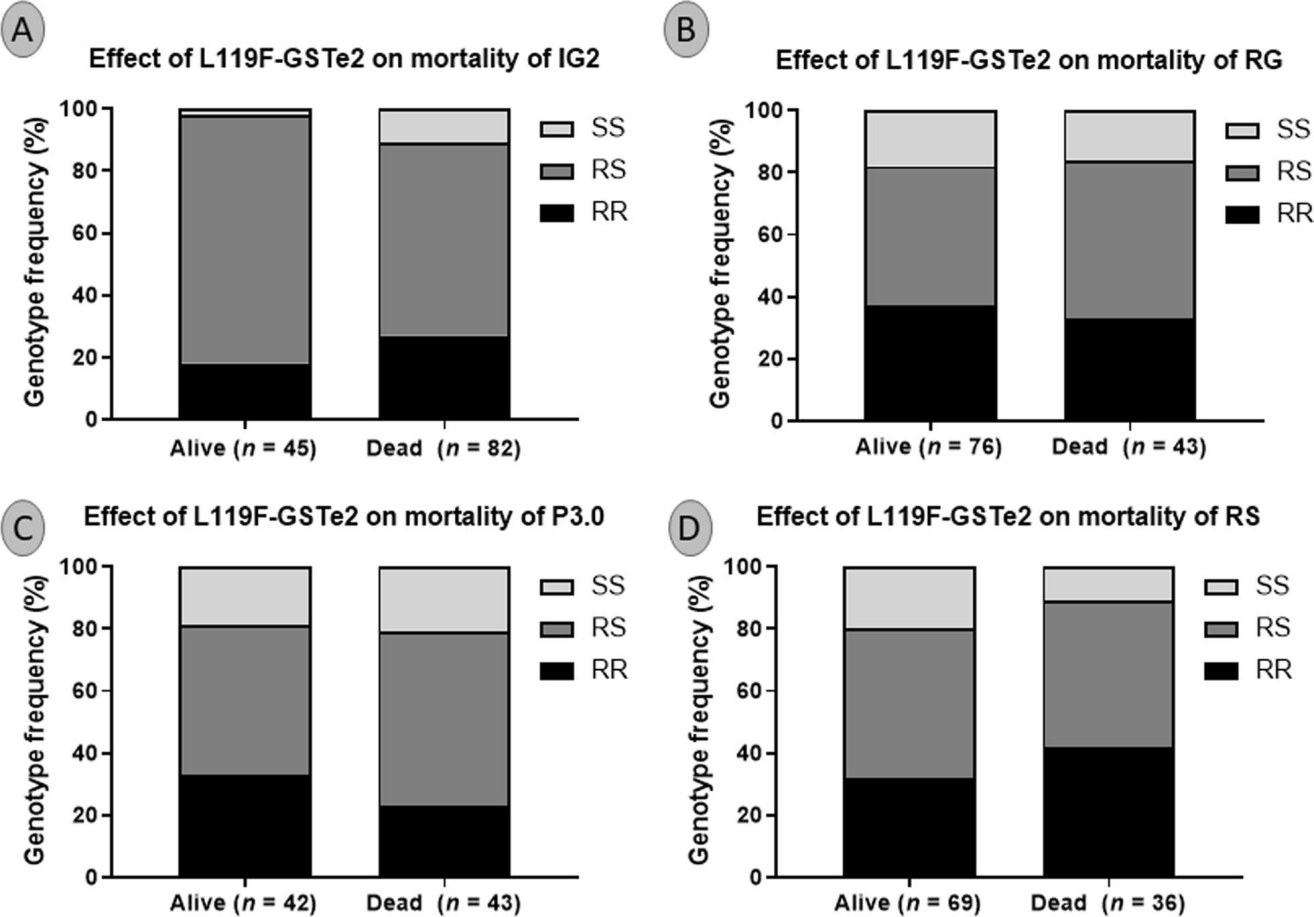
Correlation between L119F-GSTe2 resistant marker and the efficacy of bed nets in experimental hut trial. Genotype distribution of L119F mutation between alive and dead mosquitoes after exposure to Interceptor G2 (**A**), Royal Guard (**B**), Permanet 3.0 (**C**), and the pyrethroid-only net Royal Sentry (**D**). IG2: interceptor G2, RG: Royal guard, P3.0: Permanet 3.0 and RS: Royal Sentry. For genotype, RR: homozygote resistant, RS: heterozygote, SS: homozygote susceptible

To avoid confounding effects from exophily status, the distribution of the *L119F-GSTe2* genotypes was assessed only among mosquitoes collected in the room. We noticed a significant difference in the distribution of genotypes between alive and dead for Interceptor G2 (*χ*^2^ = 10.3; *P* = 0.006) with RR and SS mainly present among the dead (Fig. 7A). Evaluation of the odd ratio showed that RS had a higher ability to survive Interceptor G2 exposure than RR (*OR* = 1.9; 95% *CI*: 0.9–3.8; *P* = 0.04) and SS (*OR* = 7.0; 95% *CI*: 1.5–33.2; *P* = 0.004) (Fig. 7A). However, no difference was seen between RR and SS (*OR* = 3.6; 95% *CI*: 0.7–18.5; *P* = 0.09). At the allelic level, no difference was noted between mosquitoes harboring the 119F resistant allele and those with the L119 susceptible allele (*OR* = 1; 95% *CI*: 0.5–1.7; *P* = 0.5). Similarly, no significant difference was observed between this mutation and the ability of mosquitoes to survive to Permanet 3.0 (*χ*^*2*^ = 2.5; *P* = 0.2) (Fig. 7B), Royal Guard (*χ*^2^ = 0.7; P = 0.6) (Fig. 7C) and Royal Sentry (*χ*^2^ = 3.9; *P* = 0.1) (Fig. 7D) although the proportion of RR was slightly higher among the alive mosquitoes.

### Association between the L119F mutation on blood feeding

**Fig. 8:**
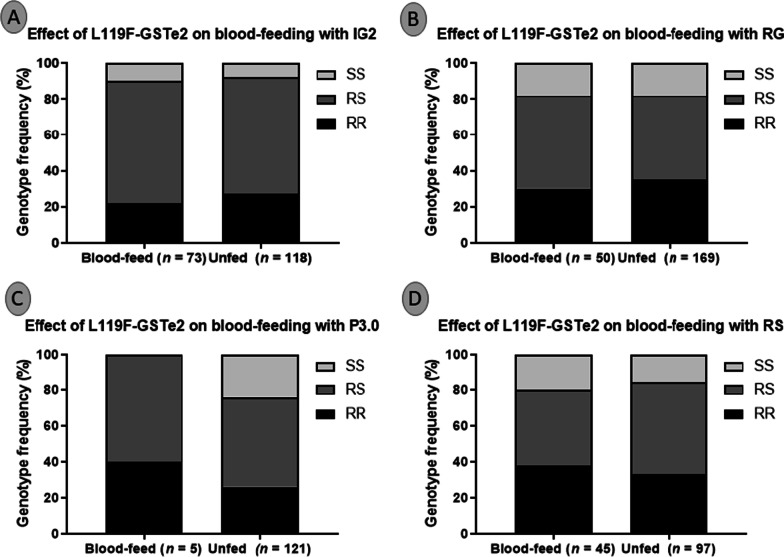
Correlation between L119F-GSTe2 resistant marker and the blood-feeding ability of field *Anopheles funestus* s.l. from Elende in the presence of various bed nets in the experimental hut trial. Genotype distribution of L119F mutation between Blood-fed and unfed mosquitoes after exposure to Interceptor G2 (**A**), Royal Guard (**B**), Permanet 3.0 (**C**), and the pyrethroid-only net Royal Sentry (**D**). IG2: interceptor G2, RG: Royal guard, P3.0: Permanet 3.0, and RS: Royal Sentry. For genotype, RR: homozygote resistant, RS: heterozygote, SS: homozygote susceptible

No association was found between the presence of the L119F mutation and the ability of mosquitoes to cross the net and take the blood of sleepers in the presence of the novel nets Interceptor G2 (*χ*^*2*^ = 0.8;* P* = 0.6) (Fig. 8A) and Royal Guard (*χ*^2^ = 0.6; *P* = 0.7) (Fig. 8B). A similar effect was observed for pyrethroid-only net Royal Sentry (*χ*^2^ = 1.6; *P* = 0.4) (Fig. 7D). In contrast, for the PBO-based net P3.0, a significant association was found between the L119F mutation and mosquitoes' blood-feeding success (*χ*^2^ = 27.9; *P* < 0.0001). Homozygote-resistant mosquitoes (RR) were significantly more likely to blood blood-fed than homozygote-susceptible (SS) (*OR* = 36.1; *P* < 0.0001) when exposed to Permanet 3.0 (Fig. 8C). Heterozygote mosquitoes (RS) also displayed significantly blood-feeding ability in the presence of Permanet 3.0 than homozygote susceptible (SS) (*OR* = 28.8 *P* < 0.0001). Homozygote-resistant mosquitoes (RR) had similar blood-feeding ability compared to heterozygote mosquitoes (RS) (*OR* = 1.3; 95% *CI*: 0.7–2.4: *P* = 0.3) (Fig. 8C). Overall, the strength of this association was further shown by the significant ability to blood feed when possessing a single 119F-resistant allele (R) compared to the susceptible allele (S) (*OR* = 100; *P* < 0.0001).

### Association between the L119F mutation and the exophily

**Fig. 9:**
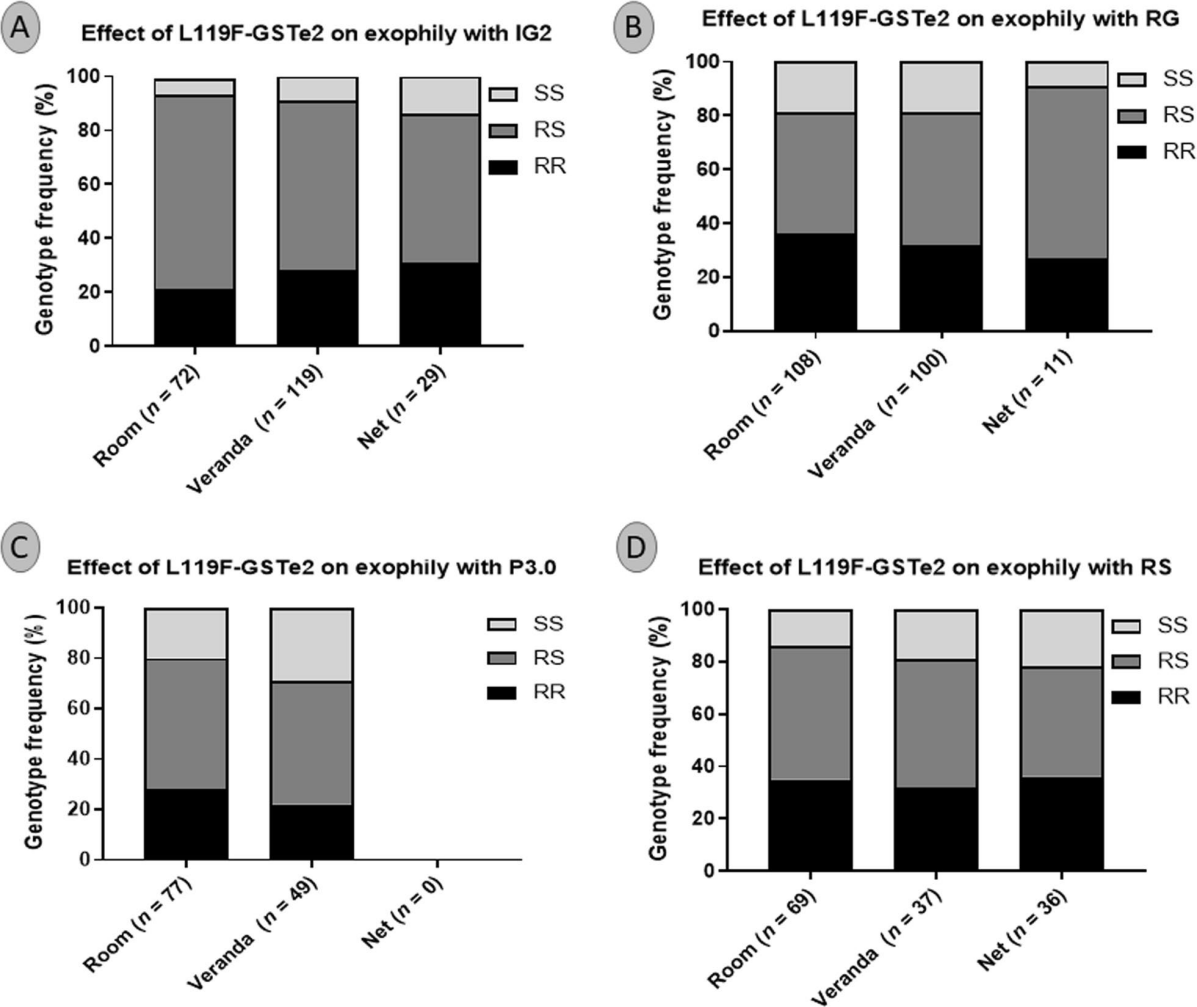
Correlation between L119F-GSTe2 resistant marker and the exiting ability of field *Anopheles funestus* s.l. from Elende in the presence of various bed nets in experimental hut trial. Genotype distribution of L119F mutation between mosquitoes collected in the room, under bednets, and in the verandah from the huts with Interceptor G2 (**A**), Royal Guard (**B**), Permanet 3.0 (**C**), and the pyrethroid-only net Royal Sentry (**D**). IG2: Interceptor G2, RG: Royal guard, P3.0: Permanet 3.0, RS: Royal Sentry. For genotype, RR: homozygote resistant, RS: heterozygote, SS: homozygote susceptible

No association was found in the distribution of L119F genotypes between mosquitoes found in the room, under the net, and those in the veranda irrespective of the bed net brand (Fig. 7A–D). For the novel net Interceptor G2, mosquitoes with RR genotype were mainly found under the net and in the verandah compared to the room but with no significant difference (*χ*^2^ = 7.6; *P* = 0.1). Same observation was seeing with Royal Guard (*χ*^2^ = 9.3; *P* = 0.05) and Royal Sentry (*χ*^2^ = 2.9; *P* = 0.6). For the PBO-based net, no mosquito was found under the net and RR was found mainly in the room whereas SS were mainly found in the verandah but still with no significant difference (*χ*^2^ = 2.5; *P* = 0.3). These results suggest that this mutation has no influence on the ability of mosquitoes to exit the veranda or to stay in the room (see Fig. 9).

## Discussion

There is an urgent need for novel alternative insecticides which can complement the pyrethroids on LLIN and improve the control of pyrethroid-resistant vectors to sustain progress against malaria. The objective of the present study was to provide evidence of the efficacy of new-generation nets against malaria vectors in Cameroon to guide NMCP in their choice for future mass distribution campaigns.

### Chlorfenapyr-based net Interceptor G2 displayed higher efficacy on field *An. funestus* s.l. from Elende in EHT

In this study, higher efficacy of the Interceptor G2 (87.7% and 57.7% mortality for unwashed and 20 washes respectively) was observed compared to the PBO-based net Permanet 3.0 (53.8% and 47.2% mortality for unwashed and 20 washes respectively), the dual AI net Royal Guard (22.13% and 17.9% mortality for unwashed and 20 washes respectively) and the pyrethroid-only net Royal Sentry (21.7% and 18.8% mortality). The low mortality response for other nets is typical of other pyrethroid-only net evaluated in Cameroon and this is attributed to insecticide resistance. *An. funestus* s.l. has developed a high-level of resistance to pyrethroids through metabolic resistance across the country which leads to the loss of efficacy of bednet as reported previously [25, 35, 36]. Because of such a high level of resistance, no difference was observed between Royal Sentry unwashed and 20-time washed (21% mortality for 0 wash and 18% over 20 washes). Similar observations were made in Benin where a mortality rate of 20% only was obtained for alphacypermethrin-only (Interceptor) net [20, 21], and in Tanzania against unwashed Interceptor and 20 times washed treatments with only 8% and 24% of host-seeking *An. gambiae* s.l. dead respectively after exposure to this net. The high efficacy of Interceptor G2 should be therefore attributed to the chlorfenapyr component in this net as reported in many African countries a full susceptibility of *An. funestus* s.l. to this insecticide [37]. This new net having a mixture of chlorfenapyr and alphacypermethrin showed also a better efficacy compared to Interceptor^®^ having only alphacypermethrin and a chlorfenapyr-only net against pyrethroid-resistant *An. gambiae* s.l. in experimental field huts in Cote d’ivoire [19].

Previous studies affirmed that chlorfenapyr was important for killing pyrethroid-resistant mosquitoes, but did not agree on the contributions of alphacypermethrin and chlorfenapyr to blood-feeding inhibition [20]. The effect of alpha-cypermethrin on blood-feeding inhibition was reported in the study with Miranet and Magnet which greatly inhibited blood-feeding compared to untreated nets, despite that it had only a limited mortality effect compared to untreated nets [38]. In our study, P3.0, Royal Guard, and Interceptor G2 0wash showed better blood-feeding inhibition and personal protection rate compared to alphacypermethrin-only net Royal Sentry. The blood-feeding inhibition of 66.2% for unwashed Interceptor G2 observed in this study is similar to other locations which suggests that chlorfenapyr could also inhibits blood-feeding in mosquitoes as reported by N’Guessan et al. [20]. The authors showed that Interceptor^®^ G2, Interceptor^®^, and chlorfenapyr net inhibited blood-feeding by 60%, 43%, and 57%, respectively. Considering the increased mortality with their use, the high personal protection rate, and their wash durability, Interceptor^®^ G2 could be an efficient replacement for pyrethroid-only ITNs for the reduction of malaria transmission in Cameroon as observed in randomised control trials in Benin and Tanzania [22, 24] although the efficacy of this net needs to be investigated in other regions across the country.

### The PBO-based net PermaNet 3.0 showed a better personal protection rate but reduced mortality rate in EHT compared to Interceptor G2

In the cone test, Permanet 3.0 induced 100% mortality in almost all the populations when using the top which contains the PBO confirming the high efficacy of this section of the Permanet 3.0net. The side part of this net induced very low mortality (< 20% in all the localities) highlighting the strong implication of P450s in pyrethroid resistance in this *Anopheles* species as reported in many other locations [25, 35, 36]. The high efficacy of Permanet 3.0 top is therefore associated with the synergist as PBO inhibits cytochrome P450 enzymes, which are key players in insecticide resistance, and also increases cuticular penetration of insecticides [39].

In EHT, Permanet 3.0 showed also a good efficacy (53.8% and 47.2% mortality for unwashed and 20 washes respectively) compared to pyrethroid-only net. This net had the highest deterrence effect and blood-feeding inhibition compared to other nets including Interceptor G2. This result was similar to observations made in Tanzania where mortality rates of 51% and 37% were obtained for Permanet 3.0 unwashed and 20 washes respectively [40]. This confirms that PBO-based nets remain efficient against *An. funestus* s.l. population although, the mortality was below the WHO threshold of 80% from EHT. Another practical example is the increased pyrethroid-susceptibility observed in *An. gambiae* s.l. from Togo when pyrethroids were combined with PBO [41]. In a study by Ketoh et al. [39], higher mortality rates and reduced blood feeding were also observed in mosquitoes that were exposed to pyrethroid-treated nets with PBO compared to those exposed to pyrethroid-only treated nets. High efficacy of PBO nets was also reported with PermaNet 3.0 (deltamethrin + PBO) [42–44], and Olyset^®^ Plus (permethrin + PBO) [25, 45] in other countries. In all these studies, nets with synergists had higher mortality rates on mosquitoes compared to their respective insecticide-only treated nets, PermaNet 2.0 (deltamethrin only) and Olyset (permethrin only). The slightly reduced efficacy of PBO net observed in this study corroborates with the previous report in northern Cameroon (Mibellon) where a mortality rate of 30.1% was obtained for the unwashed Permanet 3.0 [25]. Loss of efficacy of pyrethroid-based ITNs including Olyset^®^ Plus (low mortality rates of mosquitoes) in *An. funestus* s.l. has been reported in Mozambique [46] as observed in our study with Permanet 3.0 (53.8% and 47.2% mortality for unwashed and 20 washes respectively). These low mortality rates might be due to pyrethroid resistance intensity in study areas as observed in a different study by Corbel et al. [43]. Such loss of efficacy to PBO-based ITNs may be due to other resistant mechanisms not addressed by PBO (a cytochrome P450 inhibitor) such as metabolic resistance due to GSTs or esterases. Menze et al. [25] observed that *An. funestus* s.l. resistant to Olyset or Olyset^®^ Plus due to L119F-GSTe2 mutation had a greater blood-feeding rate compared to mosquitoes with L119 susceptible allele. This mutation was also associated with increased exophily.

While this low mortality rate from pyrethroid + synergist-based nets is alarming, we observed that Permanet 3.0 still offers a high level of personal protection (92.8%) from a mosquito bite. Oumbouke et al. [47] recently reported also that the use of VEERALIN^®^ nets, an alphacypermethrin PBO synergist net, resulted in a higher mortality rate of mosquitoes (51 vs 29%) and a greater inhibition on blood-feeding (62.6 vs 35.4%) compared to MagNet, an alphacypermethrin-only net. The study suggests that PBO does not only improve the killing effect of the insecticicide, but also potentiates the blood-feeding inhibitory effect of pyrethroids (by increasing the insecticide surface concentration) confirming the high blood-feeding inhibition recorded with Permanet 3.0 in this study (98% for Permanet 3.0 unwashed and 83% for 20washes).

### L1014F-*kdr* mutation is negatively associated with resistance to Interceptor G2 whereas the L119F-GSTe2 mutation has no major impact

In this study, a negative association was found between the *kdrw* mutation and mosquitoes' ability to survive exposure to the chlorfenapyr-based net Interceptor G2 (*χ*^2^ = 138; *P* < 0.0001) with RR mosquitoes most found in the dead. Such negative association noticed in this study could be attributed to the chlorfenapyr component in interceptor G2 as reported previously [48]. This insecticide is considered as pro-insecticide that is activated by oxidase enzymes suggesting a potential for negative cross-resistance [49] as shown recently that mosquitoes bearing the 1014F-resistant allele were more vulnerable to chlorfenapyr [37] and clothianidin [50]. The negative association observed between the *kdr*-resistant marker here and Interceptor G2 efficacy indicates that this tool could help control *kdr*-resistant mosquitoes as observed in Benin [24]. In contrast, for all the other nets, *kdr*-resistant mosquitoes were mainly found among the alive compared to dead mosquitoes confirming the loss of efficacy of these tools in field populations of *An. gambiae* s.l [12, 51–55].

We noticed a greater ability of L119F-GSTe2 heterozygotes mosquitoes to survive Interceptor G2 exposure compared to RR and SS suggesting a heterozygote advantage but more studies are needed to validate this trend. However, this would suggest that GST-based metabolic resistance could at some extent impact the efficacy of Interceptor G2. The reason why heterozygote RS would survive better remains unknown although it could be associated with the link between GST and other resistance mechanisms since mosquitoes tend to bear multiple resistance mechanisms. The same observations were reported in the northern part of Cameroon where no significant association was observed between L119F-*GSTe2* and mortality in the experimental huts for all LLINs tested [25]. All this shows that this mutation is not contributing to the reduced efficacy of alphacypermethrin and deltamethrin-based bednets despite its ability to confer DDT/permethrin resistance [31].

Moreover, no association was found between the presence of the L119F mutation and the ability of mosquitoes to cross the net and take the blood on sleepers in the presence of the novel nets Interceptor G2 as well as for the pyrethroid-only net Royal Sentry. In contrast, for the PBO-based net P3.0, a significant association was found between the L119F mutation and the mosquitoe's blood-feeding success. Such an association between the PBO nets and the blood-feeding success of mosquitoes was previously reported in *An. funestus* s.l. from Mibellon, Cameroon [25]. This was also the case for the cytochrome P450s *CYP6P9a* and *CYP6P9b* for which the resistant alleles *CYP6P9a_R* and *CYP6P9b_R* were shown, in a release-recapture EHT, to provide a greater ability to blood feed and to survive exposure to pyrethroid-only LLIN (Permanet 2.0) [13, 16, 17]. This suggests that L119F-*GSTe2* mutation could contribute to increased malaria transmission in the areas where Permanet 3.0 is implemented as every additional bite increases the chance of sporozoite being passed to the populations. This is particularly a concern as L119F-RR resistant mosquitoes have also been shown to live longer [56, 57] and to have a greater vectorial capacity to transmit *Plasmodium* [58].

One limitation of the study is that we were not able to perform the experimental hut trial in other locations to establish the efficacy of the new net interceptor G2 across the country but this will be done in the future.

## Conclusions

The results of this study in semi-field conditions show that the dual AI net Interceptor^®^ G2 and the PBO net permanet 3.0 performed better compared to the pyrethroid-only net Royal Sentry on *An. funestus* s.l. from Elende. Our work suggests that long-lasting nets with a mixture of chlorfenapyr and pyrethroids have great potential in controlling pyrethroid-resistant *An. funestus* s.l. in Cameroon followed by PBO-nets and should be urgently implemented. However, tests need to be conducted against other malaria vectors with potentially different resistant profiles and mechanisms to confirm the extent of Interceptor G2 efficacy. Furthermore, the greater mortality induced by Interceptor G2 on L1014F-*kdr* bearing mosquitoes shows that Interceptor G2 could help control now common Kdr-resistant mosquitoes.

### Supplementary Information

**Additional file 1: Figure S1.** Quality control of LLINs tested using susceptible lab strain KISUMU

**Additional file 2: Table S1.** Abundance of *An. funestus* obtained after experimental hut trials in Elende, Cameroon from December 2020 to January 2021.

**Additional file 3: Table S2a** Linear mixed effect model analysis showing different sources of variations that may influenced the exophily. **b** Linear mixed effect model analysis showing different sources of variations that may influence the blood feeding. **c** Linear mixed model effect analysis showing different sources of variations that may influenced the mortality.

## Data Availability

All the relevant datasets supporting the conclusions of this article are included within the article.

## Abbreviations

AI: Active ingredients
*CI*: Confidence interval
EHT: Experimental hut trials
IG2: Interceptor G2
ITNs: Insecticide-treated nets
kdr: Knock-down resistance
NMCP: National malaria control program
*OR*: Odd ratio
P3.0: Permanent 3.0
PBO: Piperonyl-butoxide
PIIVEC: Partnership for Increasing the Impact of Vector Control
RCTs: Randomized control trials
RG: Royal Guard
RS: Royal Sentry
TVCAG: Technical Vector Control Advisory Group
0w: Unwashed nets
20w: 20 Times-washed nets

## References

[1] Bhatt S, Weiss D, Cameron E, Bisanzio D, Mappin B, Dalrymple U, Battle K, Moyes C, Henry A, Eckhoff PJN (2015). The effect of malaria control on *Plasmodium*
*falciparum* in Africa between 2000 and 2015. Nature.

[2] Koudou B, Tano Y, Doumbia M, Nsanzabana C, Cisse G, Girardin O, Dao D, N'Goran E, Vounatsou P, Bordmann G (2005). Insecticide-treated bed nets and curtains for preventing malaria. Med Vet Entomol.

[3] Pluess B, Tanser FC, Lengeler C, Sharp BL (2010). Indoor residual spraying for preventing malaria. Cochrane Database Syst Rev.

[4] Protopopoff N, Wright A, West PA, Tigererwa R, Mosha FW, Kisinza W, Kleinschmidt I, Rowland M (2015). Combination of insecticide treated nets and indoor residual spraying in northern Tanzania provides additional reduction in vector population density and malaria transmission rates compared to insecticide treated nets alone: a randomised control trial. PLoS ONE.

[5] Lim SS, Fullman N, Stokes A, Ravishankar N, Masiye F, Murray CJ, Gakidou E (2011). Net benefits: a multicountry analysis of observational data examining associations between insecticide-treated mosquito nets and health outcomes. PLoS Med.

[6] Zaim M, Aitio A, Nakashima N (2000). Safety of pyrethroid-treated mosquito nets. Med Vet Entomol.

[7] WHO (2012). Global plan for insecticide resistance management in malaria vectors.

[8] Kelly-Hope L, Ranson H, Hemingway J (2008). Lessons from the past: managing insecticide resistance in malaria control and eradication programmes. Lancet Infect Dis.

[9] Hemingway J, Ranson H, Magill A, Kolaczinski J, Fornadel C, Gimnig J, Coetzee M, Simard F, Roch DK, Hinzoumbe CK (2016). Averting a malaria disaster: will insecticide resistance derail malaria control?. Lancet.

[10] WHO (2015). Malaria Policy Advisory Committee to the WHO: conclusions and recommendations of seventh biannual meeting (March 2015). Malar J.

[11] Ranson H, Nguessan R, Lines J, Moiroux N, Nkuni Z, Corbel V (2011). Pyrethroid resistance in African anopheline mosquitoes: what are the implications for malaria control?. Trends Parasitol.

[12] Fadel AN, Ibrahim SS, Tchouakui M, Terence E, Wondji MJ, Tchoupo M, Wanji S, Wondji CS (2019). A combination of metabolic resistance and high frequency of the 1014F kdr mutation is driving pyrethroid resistance in *Anopheles*
*coluzzii* population from Guinea savanna of Cameroon. Parasit Vectors.

[13] Weedall GD, Mugenzi LMJ, Menze BD, Tchouakui M, Ibrahim SS, Amvongo-Adjia N, Irving H, Wondji MJ, Tchoupo M, Djouaka R (2019). A cytochrome P450 allele confers pyrethroid resistance on a major African malaria vector, reducing insecticide-treated bednet efficacy. Sci Transl Med.

[14] Wamba ANR, Ibrahim SS, Kusimo MO, Muhammad A, Mugenzi LMJ, Irving H, Wondji MJ, Hearn J, Bigoga JD, Wondji CS (2021). The cytochrome P450 CYP325A is a major driver of pyrethroid resistance in the major malaria vector *Anopheles*
*funestus* in Central Africa. Insect Biochem Mol Biol.

[15] Irving H, Wondji CS (2017). Investigating knockdown resistance (kdr) mechanism against pyrethroids/DDT in the malaria vector *Anopheles*
*funestus* across Africa. BMC Genet.

[16] Mugenzi LM, Menze BD, Tchouakui M, Wondji MJ, Irving H, Tchoupo M, Hearn J, Weedall GD, Riveron JM, Cho-Ngwa F (2020). A 6.5-kb intergenic structural variation enhances P450-mediated resistance to pyrethroids in malaria vectors lowering bed net efficacy. Mol Ecol.

[17] Mugenzi LM, Menze BD, Tchouakui M, Wondji MJ, Irving H, Tchoupo M, Hearn J, Weedall GD, Riveron JM, Wondji CS (2019). Cis-regulatory CYP6P9b P450 variants associated with loss of insecticide-treated bed net efficacy against *Anopheles*
*funestus*. Nat Commun.

[18] WHO (2019). List of WHO prequalified vector control products.

[19] Camara S, Alou LA, Koffi A, Clegban Y, Kabran J-P, Koffi F, Koffi K, Pennetier C (2018). Efficacy of Interceptor® G2, a new long-lasting insecticidal net against wild pyrethroid-resistant *Anopheles gambiae* ss from Côte d’Ivoire: a semi-field trial. Parasite.

[20] N’Guessan R, Odjo A, Ngufor C, Malone D, Rowland M (2016). A Chlorfenapyr Mixture Net Interceptor® G2 shows high efficacy and wash durability against resistant mosquitoes in West Africa. PLoS ONE.

[21] Ngufor C, Fagbohoun J, Critchley J, N'Guessan R, Todjinou D, Malone D, Akogbeto M, Rowland M (2017). Which intervention is better for malaria vector control: insecticide mixture long-lasting insecticidal nets or standard pyrethroid nets combined with indoor residual spraying?. Malar J.

[22] Tungu PK, Michael E, Sudi W, Kisinza WW, Rowland M (2021). Efficacy of interceptor(R) G2, a long-lasting insecticide mixture net treated with chlorfenapyr and alpha-cypermethrin against *Anopheles*
*funestus*: experimental hut trials in north-eastern Tanzania. Malar J.

[23] Mosha JF, Kulkarni MA, Lukole E, Matowo NS, Pitt C, Messenger LA, Mallya E, Jumanne M, Aziz T, Kaaya R (2022). Effectiveness and cost-effectiveness against malaria of three types of dual-active-ingredient long-lasting insecticidal nets (LLINs) compared with pyrethroid-only LLINs in Tanzania: a four-arm, cluster-randomised trial. The Lancet.

[24] Accrombessi M, Cook J, Ngufor C, Sovi A, Dangbenon E, Yovogan B, Akpovi H, Hounto A, Thickstun C, Padonou GG (2021). Assessing the efficacy of two dual-active ingredients long-lasting insecticidal nets for the control of malaria transmitted by pyrethroid-resistant vectors in Benin: study protocol for a three-arm, single-blinded, parallel, cluster-randomized controlled trial. BMC Infect Dis.

[25] Menze BD, Kouamo MF, Wondji MJ, Tchapga W, Tchoupo M, Kusimo MO, Mouhamadou CS, Riveron JM, Wondji CS (2020). An experimental hut evaluation of PBO-based and pyrethroid-only nets against the malaria vector *Anopheles*
*funestus* reveals a loss of bed nets efficacy associated with GSTe2 metabolic resistance. Genes (Basel).

[26] Brice Natchema SF, Tchouakui M, Menze BD, Mugenzi LM, Fofie D, Nguifo-Nguete D, Nkengazong L, Tombi J, Wondji CS (2023). Entomological longitudinal surveys in two contrasted eco-climatic settings in Cameroon reveal a high malaria transmission from *Anopheles*
*funestus* associated with GSTe2 metabolic resistance. Res Sq.

[27] Tepa A, Kengne-Ouafo JA, Djova VS, Tchouakui M, Mugenzi LM, Djouaka R, Pieme CA, Wondji CS (2022). Molecular drivers of multiple and elevated resistance to insecticides in a population of the malaria vector *Anopheles*
*gambiae* in agriculture hotspot of West Cameroon. Genes.

[28] Piameu M, Nwane P, Toussile W, Mavridis K, Wipf NC, Kouadio PF, Mbakop LR, Mandeng S, Ekoko WE, Toto JC (2021). Pyrethroid and etofenprox resistance in *Anopheles*
*gambiae* and *Anopheles*
*coluzzii* from vegetable farms in Yaoundé, Cameroon: dynamics, intensity and molecular basis. Molecules.

[29] Fondjo E, Toto J-C, Tchouakui M, Eyisap WE, Patchoke S, Menze B, Njeambosay B, Zeukeug F, Ngomdjum RT, Mandeng E (2023). High vector diversity and malaria transmission dynamics in five sentinel sites in Cameroon. Malar J.

[30] World Health Organization (2013). Guidelines for laboratory and field-testing of long-lasting insecticidal nets.

[31] Riveron JM, Yunta C, Ibrahim SS, Djouaka R, Irving H, Menze BD, Ismail HM, Hemingway J, Ranson H, Albert A (2014). A single mutation in the GSTe2 gene allows tracking of metabolically based insecticide resistance in a major malaria vector. Genome Biol.

[32] Badolo A, Guelbéogo WM, Tiono AB, Traoré A, Sagnon NF, Sirima SB (2014). Laboratory evaluation of Fendona 6SC^®^ treated bednets and Interceptor^®^ long-lasting nets against *Anopheles*
*gambiae* sl in Burkina Faso. Parasitol Res.

[33] Chouaibou M, Simard F, Chandre F, Etang J, Darriet F, Hougard J-M (2006). Efficacy of bifenthrin-impregnated bednets against *Anopheles*
*funestus* and pyrethroid-resistant *Anopheles*
*gambiae* in North Cameroon. Malar J.

[34] Tchouakui M, Miranda JR, Mugenzi LM, Djonabaye D, Wondji MJ, Tchoupo M, Tchapga W, Njiokou F, Wondji CS (2020). Cytochrome P450 metabolic resistance (CYP6P9a) to pyrethroids imposes a fitness cost in the major African malaria vector *Anopheles*
*funestus*. Heredity.

[35] Menze BD, Wondji MJ, Tchapga W, Tchoupo M, Riveron JM, Wondji CS (2018). Bionomics and insecticides resistance profiling of malaria vectors at a selected site for experimental hut trials in central Cameroon. Malar J.

[36] Tchouakui M, Fossog BT, Ngannang BV, Djonabaye D, Tchapga W, Njiokou F, Wondji CS (2019). Investigation of the influence of a glutathione S-transferase metabolic resistance to pyrethroids/DDT on mating competitiveness in males of the African malaria vector, *Anopheles*
*funestus*. Wellcome Open Res.

[37] Tchouakui M, Assatse T, Tazokong HR, Oruni A, Menze BD, Nguiffo-Nguete D, Mugenzi LM, Kayondo J, Watsenga F, Mzilahowa T (2022). Detection of chlorfenapyr resistance in the malaria vector *Anopheles*
*gambiae* contrasts with full susceptibility in *Anopheles*
*funestus* across Africa. Res Sq.

[38] Oumbouke WA, Koffi AA, Alou LPA, Rowland M, N’Guessan R (2019). Evaluation of standard pyrethroid based LNs (MiraNet and MagNet) in experimental huts against pyrethroid resistant *Anopheles*
*gambiae* sl M’bé, Côte d’Ivoire: potential for impact on vectorial capacity. PLoS ONE.

[39] Ketoh GK, Ahadji-Dabla KM, Chabi J, Amoudji AD, Apetogbo GY, Awokou F, Glitho IA (2018). Efficacy of two PBO long lasting insecticidal nets against natural populations of *Anopheles*
*gambiae* s.l. in experimental huts, Kolokopé, Togo. PLoS ONE.

[40] Tungu P, Magesa S, Maxwell C, Malima R, Masue D, Sudi W, Myamba J, Pigeon O, Rowland M (2010). Evaluation of PermaNet 3.0 a deltamethrin-PBO combination net against *Anopheles*
*gambiae* and pyrethroid resistant *Culex*
*quinquefasciatus* mosquitoes: an experimental hut trial in Tanzania. Malar J.

[41] Gleave K, Lissenden N, Chaplin M, Choi L, Ranson H (2021). Piperonyl butoxide (PBO) combined with pyrethroids in insecticide-treated nets to prevent malaria in Africa. Cochrane Database Syst Rev.

[42] Birhanu A, Asale A, Yewhalaw D (2019). Bio-efficacy and physical integrity of piperonylbutoxide coated combination net (PermaNet® 3.0) against pyrethroid resistant population of *Anopheles*
*gambiae* sl and *Culex*
*quinquefasciatus* mosquitoes in Ethiopia. Malar J.

[43] Corbel V, Chabi J, Dabiré RK, Etang J, Nwane P, Pigeon O, Akogbeto M, Hougard J-M (2010). Field efficacy of a new mosaic long-lasting mosquito net (PermaNet® 3.0) against pyrethroid-resistant malaria vectors: a multi centre study in Western and Central Africa. Malar J.

[44] Awolola ST, Adeogun AO, Olojede JB, Oduola AO, Oyewole IO, Amajoh CN (2014). Impact of PermaNet 3.0 on entomological indices in an area of pyrethroid resistant *Anopheles*
*gambiae* in south-western Nigeria. Parasites Vectors.

[45] Pennetier C, Bouraima A, Chandre F, Piameu M, Etang J, Rossignol M, Sidick I, Zogo B, Lacroix M-N, Yadav R (2013). Efficacy of Olyset^®^ Plus, a new long-lasting insecticidal net incorporating permethrin and piperonil-butoxide against multi-resistant malaria vectors. PLoS ONE.

[46] Riveron JM, Huijben S, Tchapga W, Tchouakui M, Wondji MJ, Tchoupo M, Irving H, Cuamba N, Maquina M, Paaijmans K (2019). Escalation of pyrethroid resistance in the malaria vector *Anopheles funestus* induces a loss of efficacy of piperonyl butoxide-based insecticide-treated nets in Mozambique. J Infect Dis.

[47] Oumbouke WA, Rowland M, Koffi AA, Alou L, Camara S, N’Guessan R (2019). Evaluation of an alpha-cypermethrin+ PBO mixture long-lasting insecticidal net VEERALIN^®^ LN against pyrethroid resistant *Anopheles*
*gambiae* ss: an experimental hut trial in M’bé, central Côte d’Ivoire. Parasit Vectors.

[48] Tchouakui M, Assatse T, Tazokong HR, Oruni A, Menze BD, Nguiffo-Nguete D, Mugenzi LM, Kayondo J, Watsenga F, Mzilahowa T (2023). Detection of a reduced susceptibility to chlorfenapyr in the malaria vector *Anopheles*
*gambiae* contrasts with full susceptibility in *Anopheles*
*funestus* across Africa. Sci Rep.

[49] Raghavendra K, Barik TK, Sharma P, Bhatt RM, Srivastava HC, Sreehari U, Dash AP (2011). Chlorfenapyr: a new insecticide with novel mode of action can control pyrethroid resistant malaria vectors. Malar J.

[50] Tchouakui M, Assatse T, Mugenzi LM, Menze BD, Nguiffo-Nguete D, Tchapga W, Kayondo J, Watsenga F, Manzambi EZ, Osae M (2022). Comparative study of the effect of solvents on the efficacy of neonicotinoid insecticides against malaria vector populations across Africa. Infect Dis Poverty.

[51] Ibrahim SS, Muhammad A, Hearn J, Weedall GD, Nagi SC, Mukhtar MM, Fadel AN, Mugenzi LJ, Patterson EI, Irving H (2022). Molecular drivers of insecticide resistance in the Sahelo-Sudanian populations of a major malaria vector. bioRxiv.

[52] Ibrahim SS, Mukhtar MM, Irving H, Riveron JM, Fadel AN, Tchapga W, Hearn J, Muhammad A, Sarkinfada F, Wondji CS (2020). Exploring the mechanisms of multiple insecticide resistance in a highly plasmodium-infected malaria vector *Anopheles*
*funestus* Sensu Stricto from Sahel of Northern Nigeria. Genes (Basel).

[53] Mugenzi LMJ, Akosah-Brempong G, Tchouakui M, Menze BD, Tekoh TA, Tchoupo M, Nkemngo FN, Wondji MJ, Nwaefuna EK, Osae M (2022). Escalating pyrethroid resistance in two major malaria vectors *Anopheles*
*funestus* and *Anopheles*
*gambiae* (s.l.) in Atatam, Southern Ghana. BMC Infect Dis.

[54] Tchouakui M, Mugenzi LM, D. Menze B, Khaukha JN, Tchapga W, Tchoupo M, Wondji MJ, Wondji CS (2021). Pyrethroid resistance aggravation in Ugandan malaria vectors is reducing bednet efficacy. Pathogens.

[55] Tchouakui M, Oruni A, Assatse T, Manyaka CR, Tchoupo M, Kayondo J, Wondji CS (2022). Fitness cost of target-site and metabolic resistance to pyrethroids drives restoration of susceptibility in a highly resistant Anopheles gambiae population from Uganda. PLoS ONE.

[56] Tchakounte A, Tchouakui M, Mu-Chun C, Tchapga W, Kopia E, Soh PT, Njiokou F, Riveron JM, Wondji CS (2019). Exposure to the insecticide-treated bednet PermaNet 2.0 reduces the longevity of the wild African malaria vector *Anopheles*
*funestus* but GSTe2-resistant mosquitoes live longer. PLoS ONE.

[57] Tchouakui M, Riveron JM, Djonabaye D, Tchapga W, Irving H, Soh Takam P, Njiokou F, Wondji CS (2018). Fitness costs of the glutathione S-transferase epsilon 2 (L119F-GSTe2) mediated metabolic resistance to insecticides in the Major African malaria vector *Anopheles*
*funestus*. Genes (Basel).

[58] Tchouakui M, Chiang M-C, Ndo C, Kuicheu CK, Amvongo-Adjia N, Wondji MJ, Tchoupo M, Kusimo MO, Riveron JM, Wondji CS (2019). A marker of glutathione S-transferase-mediated resistance to insecticides is associated with higher *Plasmodium* infection in the African malaria vector *Anopheles funestus*. Sci Rep.

