# Supplementary figures and images for: High efficacy of chlorfenapyr-based net Interceptor® G2 against pyrethroid-resistant malaria vectors from Cameroon
Source: Infect Dis Poverty. 2023 Aug 29;12:81. doi: 10.1186/s40249-023-01132-w (PMC10463949; doi:10.1186/s40249-023-01132-w)

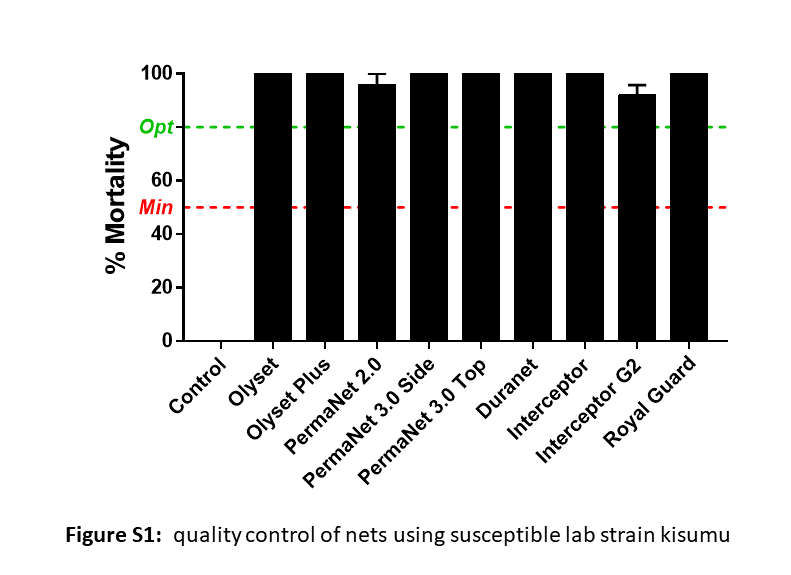

Supplement: Supplementary file 1 — Additional file 1: Figure S1. Quality control of LLINs tested using susceptible lab strain KISUMU [file 40249_2023_1132_MOESM1_ESM.png]
