# Supplementary material for: High efficacy of chlorfenapyr-based net Interceptor® G2 against pyrethroid-resistant malaria vectors from Cameroon
Source: Infect Dis Poverty. 2023 Aug 29;12:81. doi: 10.1186/s40249-023-01132-w (PMC10463949; doi:10.1186/s40249-023-01132-w)
Supplement: Supplementary file 2 — Additional file 2: Table S1. Abundance of An. funestus obtained after experimental hut trials in Elende, Cameroon from December 2020 to January 2021. [file 40249_2023_1132_MOESM2_ESM.docx]

|  |  | Control 1 | RS 20W | RS 0W | RG 20W | RG 0W | P3.0 20W | P3.0 0W | IG2 20W | IG2 0W |
| --- | --- | --- | --- | --- | --- | --- | --- | --- | --- | --- |
|  | Females Caught | 213 | 248 | 253 | 367 | 253 | 106 | 104 | 187 | 221 |
|  | Inside Room | 105 | 104 | 101 | 162 | 97 | 41 | 47 | 91 | 105 |
| Exophily | | | | | | | | | | |
|  | Inside Veranda | 82 | 108 | 136 | 195 | 150 | 58 | 57 | 70 | 105 |
|  | Inside Net | 26 | 36 | 16 | 10 | 6 | 7 | 2 | 26 | 11 |
|  | Inside Net (%) | 12.2 | 14.5 | 6.3 | 2.7 | 2.4 | 6.6 | 1.9 | 13.9 | 5.0 |
|  | Exophily (%) | 38.5 | 43.5 | 53.8 | 53.1 | 59.3 | 54.7 | 54.8 | 37.4 | 47.5 |
|  | 95% Conf. Limits | 31.9–45.1 | 37.4–49.7 | 47.6–59.9 | 48.1–58.2 | 53.2–65.3 | 45.2–64.2 | 45.2–64.4 | 30.5–44.4 | 40.9–54.1 |
|  | Induced Exophily (%) | - | NS | 24.8 | 23.8 | 33.8 | 26.4 | 26.5 | NS | NS |
| Blood feeding | | | | | | | | | | |
|  | Blood Fed | 57 | 78 | 62 | 54 | 15 | 18 | 2 | 30 | 20 |
|  | Blood Fed (%) | 26.8 | 31.5 | 24.5 | 14.7 | 5.9 | 17.0 | 1.9 | 16.0 | 9.0 |
|  | 95% Conf. Limits | 20.8–32.7 | 25.7–37.2 | 19.2–29.8 | 11.1–18.3 | 3.1–8.8 | 9.8–24.1 | –0.7–4.6 | 10.8–21.3 | 5.3–12.8 |
|  | Blood Feed Inhibition. (%) | - | NS | NS | 45.0 | 77.8 | NS | 92.8 | 40.1 | 66.2 |
|  | Blood Fed Dead | 3 | 9 | 11 | 6 | 4 | 8 | 1 | 15 | 9 |
| Mortality | | | | | | | | | | |
|  | Overall Mortality 72h | 13 | 54 | 46 | 66 | 56 | 50 | 56 | 104 | 194 |
|  | Overall Mortality (%) | 6.1 | 21.8 | 18.2 | 18.0 | 22.1 | 47.2 | 53.8 | 55.6 | 87.8 |
|  | 95% Conf.Limits | 2.9–9.3 | 16.6–26.9 | 13.4–22.9 | 14.1–21.9 | 17.1–27.2 | 37.7–56.7 | 44.3–63.4 | 48.5–62.7 | 83.5–92.1 |
|  | Immediate Mortality | 6 | 35 | 19 | 38 | 27 | 40 | 43 | 76 | 87 |
|  | Immediate Mort. / Total (%) | 2.8 | 14.1 | 7.5 | 10.3 | 10.7 | 37.7 | 41.3 | 40.6 | 39.4 |

**Table S1:** Abundance of *An funestus* obtained after experimental hut trials in Elende. Cameroon from December 2020 to January 2021

**RS**= Royal Sentry (pyrethroid-only); **RG**=Royal Guard (pyrethroid+ pyriproxifen); **IG2**= Interceptor G2 (pyrethroid+chlorfenapyr); **P3.0**= Permanet 3.0 (pyrethroid+PBO). NS= not applicable
