# Supplementary material for: High efficacy of chlorfenapyr-based net Interceptor® G2 against pyrethroid-resistant malaria vectors from Cameroon
Source: Infect Dis Poverty. 2023 Aug 29;12:81. doi: 10.1186/s40249-023-01132-w (PMC10463949; doi:10.1186/s40249-023-01132-w)
Supplement: Supplementary file 3 — Additional file 3: Table S2a Linear mixed effect model analysis showing different sources of variations that may influenced the exophily. b Linear mixed effect model analysis showing different sources of variations that may influence the blood feeding. c Linear mixed model effect analysis showing different sources of variations that may influenced the mortality. [file 40249_2023_1132_MOESM3_ESM.docx]

**Table S2a.** Linear mixed effect model analysis showing different sources of variations that may influenced the exophily.

|  | **Estimate** | **Std. Error** | ***t* value** | *P*-value | **Significance**  **level** |
| --- | --- | --- | --- | --- | --- |
| **(Intercept)** | 3.26849 | 0.309895 | 10.547 | < 2e-16 | *** |
| **Interceptor G20wash** | 0.277137 | 0.226463 | 1.224 | 0.2223 | ns |
| **Interceptor G220wash** | 0.06642 | 0.235563 | 0.282 | 0.7782 | ns |
| **Permanet 3.0 0wash** | 0.25726 | 0.23254 | 1.106 | 0.2697 | ns |
| **Permanet 3.0 20wash** | 0.511968 | 0.216609 | 2.364 | 0.0189 | * |
| **Royal Guard 0wash** | 0.403497 | 0.213738 | 1.888 | 0.0603 | ns |
| **Royal Guard 20wash** | 0.408902 | 0.218318 | 1.873 | 0.0623 | ns |
| **Royal Sentry 0wash** | 0.427903 | 0.219811 | 1.947 | 0.0528 | ns |
| **Royal Sentry 20wash** | 0.21427 | 0.22182 | 0.966 | 0.3351 | ns |
| **Sleeper2** | 0.261375 | 0.208682 | 1.253 | 0.2116 | ns |
| **Sleeper3** | 0.276761 | 0.211958 | 1.306 | 0.1929 | ns |
| **Sleeper4** | 0.03176 | 0.234306 | 0.136 | 0.8923 | ns |
| **Sleeper5** | 0.137843 | 0.230552 | 0.598 | 0.5505 | ns |
| **Sleeper6** | -0.036493 | 0.236967 | -0.154 | 0.8777 | ns |
| **Sleeper7** | -0.081703 | 0.243991 | -0.335 | 0.738 | ns |
| **Sleeper8** | 0.123711 | 0.220418 | 0.561 | 0.5752 | ns |
| **Sleeper9** | 0.046823 | 0.217872 | 0.215 | 0.83 | ns |
| **Hut2** | -0.159771 | 0.243135 | -0.657 | 0.5117 | ns |
| **Hut3** | -0.200984 | 0.230382 | -0.872 | 0.3839 | ns |
| **Hut4** | -0.083343 | 0.251768 | -0.331 | 0.7409 | ns |
| **Hut5** | -0.040315 | 0.236254 | -0.171 | 0.8647 | ns |
| **Hut6** | 0.10534 | 0.225911 | 0.466 | 0.6414 | ns |
| **Hut7** | -0.243463 | 0.243999 | -0.998 | 0.3194 | ns |
| **Hut8** | -0.291107 | 0.235426 | -1.237 | 0.2175 | ns |
| **Hut9** | 0.022212 | 0.233999 | 0.095 | 0.9245 | ns |
| **Week2** | 0.117325 | 0.192375 | 0.61 | 0.5425 | ns |
| **Week3** | 0.008811 | 0.199792 | 0.044 | 0.9649 | ns |
| **Week4** | 0.266231 | 0.189444 | 1.405 | 0.1613 | ns |
| **Week5** | 0.450075 | 0.186302 | 2.416 | 0.0165 | * |
| **Week6** | 0.432687 | 0.182094 | 2.376 | 0.0183 | * |

**Table S2b.** Linear mixed model effect analysis showing different sources of variations that may influenced the Blood feeding.

|  | **Estimate** | **Std. Error** | ***t* value** | ***P*-Value** | **Significance level** |
| --- | --- | --- | --- | --- | --- |
| **(Intercept)** | 4.17065 | 0.36373 | 11.466 | < 2e-16 | *** |
| **Interceptor G20wash** | -0.95153 | 0.42358 | -2.246 | 0.025619 | * |
| **Interceptor G220wash** | -0.74553 | 0.38658 | -1.929 | 0.055011 | ns |
| **Permanet 3.0 0wash** | -2.24953 | 0.75705 | -2.971 | 0.003276 | ** |
| **Permanet 3.0 20wash** | -0.58485 | 0.37131 | -1.575 | 0.116598 | ns |
| **Royal Guard 0wash** | -0.79744 | 0.36931 | -2.159 | 0.031853 | * |
| **Royal Guard 20wash** | 0.01302 | 0.32937 | 0.04 | 0.968511 | ns |
| **Royal Sentry 0wash** | 0.19341 | 0.31976 | 0.605 | 0.54587 | ns |
| **Royal Sentry 20wash** | -0.05815 | 0.30379 | -0.191 | 0.848361 | ns |
| **Sleeper2** | -0.06311 | 0.33963 | -0.186 | 0.852758 | ns |
| **Sleeper3** | -0.61858 | 0.40857 | -1.514 | 0.131384 | ns |
| **Sleeper4** | -0.08634 | 0.34745 | -0.249 | 0.803964 | ns |
| **Sleeper5** | -0.15494 | 0.3453 | -0.449 | 0.654055 | ns |
| **Sleeper6** | -0.16349 | 0.3375 | -0.484 | 0.628554 | ns |
| **Sleeper7** | -0.39516 | 0.37041 | -1.067 | 0.287167 | ns |
| **Sleeper8** | -0.74005 | 0.40141 | -1.844 | 0.066512 | ns |
| **Sleeper9** | -0.51576 | 0.35452 | -1.455 | 0.147069 | ns |
| **Hut2** | 0.16004 | 0.37797 | 0.423 | 0.67238 | ns |
| **Hut3** | 0.10922 | 0.37345 | 0.292 | 0.770195 | ns |
| **Hut4** | 0.04887 | 0.41914 | 0.117 | 0.90728 | ns |
| **Hut5** | 0.56574 | 0.38291 | 1.477 | 0.140905 | ns |
| **Hut6** | 0.2531 | 0.38704 | 0.654 | 0.513793 | ns |
| **Hut7** | 0.03763 | 0.41222 | 0.091 | 0.927352 | ns |
| **Hut8** | -0.38313 | 0.42039 | -0.911 | 0.36305 | ns |
| **Hut9** | 0.07849 | 0.38586 | 0.203 | 0.838993 | ns |
| **Week2** | -0.69155 | 0.26851 | -2.576 | 0.010629 | * |
| **Week3** | -1.19667 | 0.33065 | -3.619 | 0.000363 | *** |
| **Week4** | -0.77109 | 0.28519 | -2.704 | 0.007363 | ** |
| **Week5** | -0.8913 | 0.30215 | -2.95 | 0.003505 | ** |
| **Week6** | -0.93357 | 0.29486 | -3.166 | 0.001752 | ** |

**Table S2c.** Linear mixed effect model analysis showing different sources of variations that may influenced the mortality.

|  | **Estimate** | **Std. Error** | ***t* value** | ***P*-Value** | **Significance level** |
| --- | --- | --- | --- | --- | --- |
| **(Intercept)** | 2.759006 | 0.412511 | 6.688 | 1.67E-10 | *** |
| **Interceptor G2 0wash** | 1.738053 | 0.296385 | 5.864 | 1.54E-08 | *** |
| **Interceptor G2 20wash** | 1.42083 | 0.310418 | 4.577 | 7.68E-06 | *** |
| **Permanet 3.0 0wash** | 1.464517 | 0.316443 | 4.628 | 6.14E-06 | *** |
| **Permanet 3.0 20wash** | 1.261111 | 0.314849 | 4.005 | 8.34E-05 | *** |
| **Royal Guard 0wash** | 0.766437 | 0.326913 | 2.344 | 0.0199 | * |
| **Royal Guard 20wash** | 0.813949 | 0.32324 | 2.518 | 0.0125 | * |
| **Royal Sentry 0wash** | 0.421015 | 0.352148 | 1.196 | 0.2331 | ns |
| **Royal Sentry 20wash** | 0.303946 | 0.355815 | 0.854 | 0.3939 | ns |
| **Sleeper2** | -0.10323 | 0.237023 | -0.436 | 0.6636 | ns |
| **Sleeper3** | -0.078557 | 0.244546 | -0.321 | 0.7483 | ns |
| **Sleeper4** | -0.007766 | 0.248647 | -0.031 | 0.9751 | ns |
| **Sleeper5** | -0.140416 | 0.271841 | -0.517 | 0.606 | ns |
| **Sleeper6** | -0.158529 | 0.261053 | -0.607 | 0.5443 | ns |
| **Sleeper7** | -0.281373 | 0.277337 | -1.015 | 0.3114 | ns |
| **Sleeper8** | -0.172367 | 0.24921 | -0.692 | 0.4898 | ns |
| **Sleeper9** | -0.147064 | 0.239515 | -0.614 | 0.5398 | ns |
| **Hut2** | -0.083302 | 0.326408 | -0.255 | 0.7988 | ns |
| **Hut3** | 0.078364 | 0.292284 | 0.268 | 0.7889 | ns |
| **Hut4** | -0.049847 | 0.328729 | -0.152 | 0.8796 | ns |
| **Hut5** | -0.25685 | 0.303003 | -0.848 | 0.3975 | ns |
| **Hut6** | -0.082264 | 0.288347 | -0.285 | 0.7757 | ns |
| **Hut7** | -0.134668 | 0.282366 | -0.477 | 0.6339 | ns |
| **Hut8** | -0.101048 | 0.271369 | -0.372 | 0.71 | ns |
| **Hut9** | -0.069187 | 0.294309 | -0.235 | 0.8144 | ns |
| **Week2** | 0.201018 | 0.206939 | 0.971 | 0.3324 | ns |
| **Week3** | 0.099291 | 0.210374 | 0.472 | 0.6374 | ns |
| **Week4** | 0.147238 | 0.215673 | 0.683 | 0.4955 | ns |
| **Week5** | -0.066971 | 0.232037 | -0.289 | 0.7731 | ns |
| **Week6** | -0.022872 | 0.222296 | -0.103 | 0.9181 | ns |
